# Expanding salivary biomarker detection by creating a synthetic neuraminic acid sensor via chimeragenesis

**DOI:** 10.1128/aem.02027-25

**Published:** 2026-04-29

**Authors:** Samuel J. Verzino, Sharona A. Priyev, Valeria A. Sánchez Estrada, Ali GholamianMogaddan, Gemma X. Crowley, Alexandra Rutkowski, Amelia C. Lam, Elizabeth S. Nazginov, Paola Kotemelo, Agustina Bacelo, Jack D. Flannery, Ksenya Gavrilov, Desiree T. Sukhram, Frank X. Vázquez, Javier F. Juárez

**Affiliations:** 1 Department of Biological Sciences, Saint John’s University, Queens, New York, USA; 2 Division of HIV, ID and Global Medicine, University of California San Francisco (UCSF) 8785, San Francisco, California, USA; 3 Department of Chemistry, Saint John’s University, Queens, New York, USA; Danmarks Tekniske Universitet, Kgs. Lyngby, Denmark

**Keywords:** whole-cell biosensor, N-acetylneuraminic, transcription factor, synthetic

## Abstract

Whole-cell biosensors (WCBs) can leverage the physiology of engineered bacteria *in vivo* to detect target molecules of interest. Detection of these analytes via minimally invasive sampling of biological fluids such as saliva will improve early detection and prognosis for a multitude of conditions. Our target compound is N-acetylneuraminic acid (Neu5Ac), associated with dysbiosis, inflammation, and oral squamous cell carcinoma. In this work, we present biosensor strain MG-Sphnx expressing the custom-made chimeric transcription factor Sphnx (LacI-LNK2-SiaP), which, upon binding Neu5Ac at physiopathological concentrations, enables the expression of a fluorescent reporter. We describe the construction and testing of a collection of chimeric transcriptional repressors, assay their functionality, characterize Sphnx among them, and test it with contrived samples. We also compared models of the 3D structure of functional and non-functional chimeras to widen our knowledge on the behavior of modular proteins. We expect these results to become the foundation of a platform for the screening of a panel of biomarkers with an array of WCBs, bringing attention to their potential for point-of-care and at-home diagnostics.

**IMPORTANCE:**

Whole-cell biosensors based on transcription factors are powerful tools with an enormous potential for point-of-care diagnostics, especially when designed to detect biomarkers from easy-to-extract biological fluids such as saliva. Detection of dysbiosis and cancer salivary biomarker N-acetylneuraminic acid (Neu5Ac) will improve patient prognosis. A common problem when designing biosensors for compounds such as Neu5Ac is the lack in the literature of a transcription factor capable of detecting the analyte of interest. Here, we describe a straightforward approach for the creation of a whole-cell biosensor based on a synthetic allosteric transcription factor capable of detecting Neu5Ac. We provide a scaled-down, affordable pipeline for the construction of a candidate library; we also characterize a candidate transcription factor and validate it with contrived salivary samples. Finally, we compare a functional and a non-functional synthetic transcription factor from our collection, hypothesizing on what are the structural differences influencing their functionality.

## INTRODUCTION

Biosensors are analytical devices that use biological components to convert a physicochemical stimulus into a detectable signal (1, 2). Specifically, whole-cell biosensors (WCBs) are detection systems in which physiologically active cells sustain an array of genetically encoded modules (receptors, transporters, enzymes, regulators, etc.) capable of signal recognition, integration, and production of an observable output (1). As such, these devices stand out due to their versatility and engineering potential. Among the organisms that can serve as WCBs, microorganisms, which either produce or limit the production of reporter proteins in the presence of a target analyte, have demonstrated their utility for environmental monitorization, food safety, medicine, and more (3, 4). Microorganisms can be grown rapidly in relatively affordable media and produce all the necessary biological elements for analyte detection, providing a cost-effective and portable alternative to other detection systems (5). Unlike conventional sensors that may require large and complex instruments as well as laborious sample processing, WCBs can detect a bioavailable substance directly from the environment with minimal preparation, enabling their incorporation into *in situ* detection hardware (5). Multiplexing strategies enabling the detection of several signals simultaneously, combined with improved safety controls (i.e., biocontainment strategies), increase the practicality of these biosensors to be deployed in both clinical and diagnostic settings (4, 6). Additionally, the robust, adaptable, and diverse nature of bacterial biosensors makes them suitable for use in environments where other technologies would not be able to operate, such as under strenuous pH and osmotic conditions (4). Bacterial chassis with extremely versatile metabolisms, such as *Pseudomonas putida KT2440,* have become powerful platforms that can be tuned to detect a wide array of chemicals (7). Engineered members of the microbiota could be used as biotherapeutic agents capable not only of detecting a biomarker but also of responding by releasing biopharmaceutical molecules, a prospect that has been explored with, for example, members of the oropharyngeal and intestinal microbiota (8). While the use of WCBs is not exempt from potential drawbacks—slow reaction times, potentially impractical dynamic ranges, and sensitivity and selectivity issues—multiple solutions have been proposed. Among them, we can find the deployment of potentiometric WCBs based on immobilized bacteria that have been shown to operate in a time scale of minutes (9), and procedures for reporter optimization that can improve reaction time, reduce the limit of detection, and improve sensitivity (10–12).

WCBs based on transcription factors (TFs) rely on these regulators interacting with their soluble ligand in such a way that this interaction becomes physically associated with a conformational change of the TF, so that its binding state to a DNA operator gets altered, modifying the transcription profile of a regulated reporter (13). Many of these TFs are monogenic transcriptional repressors (14, 15), regulators encoded by a single gene whose product prevents transcription in the absence of a small soluble inducer molecule. Native TFs shaped by millions of years of evolution to detect known target molecules can be harnessed in the laboratory for analyte detection. However, even though the catalog of characterized TFs is expanding, it is still limited, restricting biosensor construction (16, 17). Regulator customization and *de novo* creation using synthetic biology tools offer a solution to expand the panoply of molecules that can be detected (18, 19). Some efforts have focused on developing a T7-RNA polymerase-based platform for targeted expression in prokaryotes, providing avenues for regulator customization (20). New synthetic TFs have enabled the detection of molecules such as glucose (SLCP_GL_^21^) and benzoate (ChTFBz01/02 (18) in engineered bacteria, or S-adenosylmethionine (MetJ-hER-VP16 (22) in yeast. An orthogonal approach consisting of the creation of synthetic two-component systems, based on gene fusions to EnvZ to leverage the EnvZ-OmpR signal transduction system (23), has been implemented for the detection of ribose (Trg-EnvZ) (24) and aspartic acid (Tar-EnvZ) (25). In this paper, we focused on the detection of a salivary biomarker, creating an *à-la-carte* monogenic synthetic regulator.

Our interest in salivary biomarker detection is motivated by the vast array of metabolites informative of oral and systemic health contained in human saliva (26). Diagnostic screenings that do not require invasive procedures are preferred by patients, which makes saliva a prime biological fluid candidate to be sampled for health and disease markers (27, 28). The information density of saliva makes frequent or continuous sampling by portable and wearable equipment desirable, contributing to the role of dental practices at the forefront of early disease prevention and diagnosis (29), and improving at-home health monitoring (30). This approach is especially relevant due to the widespread acceptance and use of consumer technology within society, which has normalized wearable biosensors connected to a phone or a smartwatch, as well as at-home sample collection (31). Electrochemical intraoral biodevices capable of detecting salivary biomarkers presently exist, yet they are limited in the scope of molecules they detect and lack adaptability to sense new ones (32). WCBs based on engineered genetic circuits offer a plug-and-play potential, in which the substitution of one TF by another, plus its cognate operator, is often enough to repurpose a pre-existing sensor.

In this work, we focused our attention on N-acetyl-D-neuraminic acid (Neu5Ac), the most common of the sialic acids (33–35). Neu5Ac fulfills key roles in human physiology (36), as well as in commensal and pathogenic bacteria (33). This particular sialic acid is also a metabolite of industrial interest for the production of dietary supplements and pharmaceutical products, and a strong candidate for bioproduction in *Escherichia coli* and *Bacillus subtilis chassis* (37). Moreover, variations in salivary sialylation have been correlated to periodontal disease (PD) and oral cancer (38). The oral microbiome of patients with PD includes *Tannerella forsythia*, which engages in glycan harvesting behavior, cleaving Neu5Ac from glycoproteins to stealthily evade their host’s immune system (39). Abnormally high concentrations of Neu5Ac have been associated with the onset of oral cancer (40–42): a group of neoplasms that affect any region in the oral cavity, pharynx, and salivary glands (43), among which oral squamous cell carcinoma (OSCC) accounts for more than 90% of cases (43). As one of the most prevalent cancers in the world (44), untreated OSCC has a very poor prognosis and, as such, early detection is essential for successful treatment (45). The Neu5Ac concentration range identified in the saliva of healthy adults was approximately 21.65 ± 5.71 mg/dL (0.52–0.88 mM), while confirmed oral pre-cancer individuals displayed 59.75 ± 7.29 mg/dL (1.7–2.2 mM) and histopathologically confirmed OSCC patients showed 204.85 ± 60.38 mg/dL (4.6–8.6 mM), consistently superior to the other two groups (46). Independent studies also found that free sialic acid levels were significantly elevated in patients diagnosed with oral cancer (47). Under the light of these observations, salivary Neu5Ac concentration has been postulated as a biomarker for OSCC, and thus a candidate target for the construction of a biosensor. However, it is important to note that there are no current standard analysis tools for quantification of Neu5Ac in bodily fluids, despite the existence of ample literature describing colorimetric, enzymatic, and chromatographic methods for the detection of this compound (e.g., [48–50]).

Before describing the construction of a Neu5Ac WCB based on the custom-made TF object of this work, it is worth mentioning that there are examples of regulators capable of interacting with Neu5Ac. NanR, a member of the GntR (HTH) superfamily of transcriptional repressors, was originally described in *E. coli*, where it directly regulates the operon responsible for the catabolism of sialic acids (51, 52) and has been postulated to play a role as a global regulator (53). *E. coli*’s NanR behaves allosterically and possesses a Kd of 16 μM (54). Potential homologs have been found in other gram negatives (55, 56), whereas a regulator of the same name and function in *Clostridium perfringens* belongs to a different family (RpiR, wHTH) (57). Despite the existence of these regulators, we pursued the construction of a Neu5Ac-sensing TF *de novo*, with the goal of deepening our understanding of modular protein assembly. We initiated the construction of custom Neu5Ac-sensing regulators based on streamlined gene fusion methods developed by our laboratory (18). We favor the assembly of monogenic transcriptional repressors due to their simplicity, compact nature, and ease of integration into synthetic circuits (14, 15, 58). Here, we describe the construction of a Neu5Ac-induced TF, Sphnx (LacI-LNK2-SiaP), integrated into an *Escherichia coli* MG1655 strain. These metabolically active bacteria can intake a Neu5Ac signal by expressing Sphnx, which bridges detection and processing (a common feature of bioreceptors (31). A separate reporter plasmid completes the system, providing a gene encoding a fluorescent protein (sfGFP, GFP from hereon) whose expression is regulated by the synthetic TF Sphnx so that detectable changes in fluorescence occur upon the detection of soluble Neu5Ac. Sphnx is a member of a new combinatorial library of TFs assembled via a simplified chimeragenesis process. Clues on inter-domain communication of modular proteins have started to be gathered by comparing Sphnx to non-functional members of the TF collection. Protein tertiary structure modeling of functional and non-functional regulators has contributed to establish comparisons between them, helping to elucidate intricate and frequently difficult-to-predict dynamics that may condition the success or failure of any chimeric TF.

## RESULTS AND DISCUSSION

### Design and construction of synthetic transcription factors

The functionality of the WCBs designed in this work hinged on the obtention of a TF capable of binding Neu5Ac with efficacy and specificity. The Neu5Ac-induced monogenic transcriptional repressor we intended to assemble included three modules: an N-terminus (N_t_) DNA-binding domain (DBD), a linker sequence (LNK), and a C-terminus (C_t_) ligand-dinding domain (LBD). The DBD specifically recognizes an operator sequence in the promoter it regulates, driving the expression of the reporter gene (*sfgfp*). The LBD is tasked with the recognition of Neu5Ac, providing specificity to the TF. Finally, the LNK sequence acts as a bridge, providing the right amount of flexibility/rigidity necessary for the transfer of information between DBD and LBD: steric changes in the LBD associated with Neu5Ac binding get translated into steric changes in the DBD effecting a change of its oligomeric state that results in the unbinding of the TF from its operator and thus in a de-repression of transcription. By using a gene fusion/chimeragenesis approach, we generated a library of repressors with a common N_t_-DBD-LNK-LBD-C_t_ architecture.

Our selected DBD was sourced from what is possibly the most studied of transcriptional regulators: the *lac* repressor or LacI (59). We exclusively employ the DBD of LacI to recognize the operator *lacO* present in the *P_lac_* promoter (as defined in (18). We decided to use DBD-LacI because the *lac* repressor originated as a modular protein with two domains and has been previously used in the construction of several functional chimeras (21, 60, 61). It is important to note that the exclusive use of DBD-LacI, as opposed to the full protein, ensures that chimeric proteins carrying this domain will recognize their target operator, yet make them incapable of responding to native inducers (lactose/IPTG).

Conversely, since the LBD was the domain that would recognize the molecule of interest, it could be sourced from any whole protein or protein domain as opposed to being co-opted from a previously characterized regulator. Periplasmic-binding proteins (PBPs) are one of our main sources for the LBDs we employ in our chimeragenesis pipeline (62). These are standalone subunits of transporters that act as molecular shuttles that assist in the traffic of small solutes from the outer to the inner membrane in gram-negative bacteria (63). PBPs share a common ancestor with the LBD domains of the LacI/GalR family of regulators (61). To build the synthetic TF we required for this project, we needed to identify LBD candidates that strongly bound our soluble molecule of interest (Neu5Ac). PBPs SatA and SiaP were selected for their binding affinity and specificity for the transport of Neu5Ac. SatA is a product of the *satABCD* (*s*ialic *a*cid *t*ransport) operon that encodes an ATP-binding cassette (ABC) transporter (64) from *Haemophilus ducreyi* (65). An alternative *satABCD* system present in *Corynebacterium glutamicum* has been demonstrated to be essential for its growth using Neu5Ac as the sole carbon source (66). SiaP is a product of the *siaPQM* operon, which encodes a Tripartite ATP-independent periplasmic (TRAP) transporter in *Haemophilus influenzae* (67, 68). SiaP was the first TRAP-associated PBP whose structure was elucidated (in *Vibrio cholerae*, *Pasteurella multocida,* and *Fusobacterium nucleatum* (67, 69). Structural comparisons and thermodynamic studies of SatA and SiaP suggest that similar affinities for Neu5Ac are achieved in the two PBPs through distinct mechanisms: one enthalpically driven and the other entropically driven. SiaP, as well as other TRAP transporters, is hypothesized to command an enthalpically driven ligand binding strategy due to the abundance of charged residues (Arg, Lys, Asp, and Glu) at their binding pocket. SatA, alternatively, is hypothesized to bind its substrate in what is considered an entropic approach because of the numerous hydrophobic and polar residues (Tyr, Phe, Gly, Leu, Asn, and Ser) at its binding pocket (70). The structure of *H. influenzae* SiaP in the presence of Neu5Ac (PDB 3B50) reveals the ligand bound in a deep cavity with its carboxyl group forming a salt bridge with a highly conserved Arg residue (67). Initial studies of SiaP isolated from *H. influenzae* displayed a binding *K_d_* of ≈120 nM when affinity for Neu5Ac was tested (68). Subsequent studies established a value closer to *K_d_* ≈58 nM (67). Similar binding affinity of SatA complexes has been identified with a *K_d_* ≈133 nM (70). The collection of empirical data supporting the ability of SatA and SiaP to bind Neu5Ac made them ideal candidates to become the LBD of a chimeric TF responsive to Neu5Ac.

The final piece of the chimeric gene puzzle consisted of establishing a connection between DBD and LBD domains. This task was performed by the linkers (LNK), protein regions that tend to be less structured than the domains they connect (71). To maximize the chances of transmitting the information of Neu5Ac binding to the DBD, so that it can detach from *lacO* and enable transcription, we decided to connect DBD and LBD via two different strategies: (i) a direct linkage without a polypeptide linker (condition identified as LNK1 for uniformity purposes), or (ii) by one out of four linkers characterized by having different biophysical properties (identified as LNK2 to LNK5; Table S1). It is important to note that while LNK1 does not contain a linker *per se*, the LBDs we chose, by virtue of being PBPs, are proteins that would be exported to the periplasm of the cell under natural circumstances, and thus contain a native signal peptide at their N-terminus. This kind of peptide appears to be unstructured for the most part, barring a short alpha-helix region (72). We decided to include the signal peptides of both SiaP and SatA due to existing examples of functional chimeras containing similar structures, such as the glucose-inducible SLCP_GL_ (21) and the benzoate-inducible CbnR-ABE44898-OD/ChTFBz01 (18) synthetic TFs. At this stage, we had identified all the elements we needed to start assembling our new chimeric TFs.

**TABLE 1:** Chimeric transcription factors discussed in this work

Name		TF behavior		Comments
Common	Systematic	Repressor	De-repressible	
Siren	LacI-LNK1-SiaP	Yes	Yes	Functional only in late stationary phase
Sphnx	LacI-LNK2-SiaP	Yes	Yes	Biosensor candidate
Kunst	LacI-LNK3-SiaP	Yes	No	Non-functional
Scorpio	LacI-LNK4-SiaP	No	No	Non-functional
Bastet	LacI-LNK5-SiaP	No	No	Repression in early hours of growth
Lumos	LacI-LNK6-SiaP	No	No	Duplicated sequence acts as a linker
Harpy	LacI-LNK1-SatA	Yes	Unreliable	Unstable strain
Haus	LacI-LNK2-SatA	No	No	Non-functional

dsDNA fragments encoding the DBD and all LNK-LBD variants were obtained as detailed in Materials and Methods. To initiate the construction of a library containing all the desired chimeric TFs, we performed several scarless assembly reactions with all the domain-encoding fragments and linearized expression vector pUC19RBS, followed by transformation in NEB5-alpha competent cells. The resulting clones were isolated and individually sequenced. The cloning of each individual chimera was attempted five times to complete the library. Constructions that were not successful at that point were shelved. Successful chimeras cloned in pUC19RBS moved forward to the next stage, subcloning into the low-copy vector pCKTRBS. The resulting set of plasmids was collectively identified as pCKT-*Chimera*. We hypothesized that chimeras unsuccessfully cloned at this stage may be potentially deleterious for the cells. It is important to note that the expression of any heterologous protein is *per se* taxing on bacterial metabolism, and that TFs might be especially burdensome (73, 74). Chimeric constructions including DBD-LacI and SiaP/SatA might possibly provoke a certain level of toxicity to the cell, albeit to different degrees depending on the specific domain combination. Indirect evidence supporting this hypothesis was observed when constructions expressing the chimeric TFs were transformed into *E. coli* MC4100 to assess their behavior in M63 minimal media with glycerol as the sole carbon source. Under these resource-limited conditions, stressed bacteria have more difficulties in thriving, and MC4100 cells bearing pCKT-*Chimera* vectors displayed a very modest and erratic growth consistent with our assumptions (data not shown). Table 1 contains the chimeric TFs built, assayed, and presented in this study, as well as a simplified interpretation of their functionality when grown in rich media.

### Screening of transcription factors

**Fig 1:**
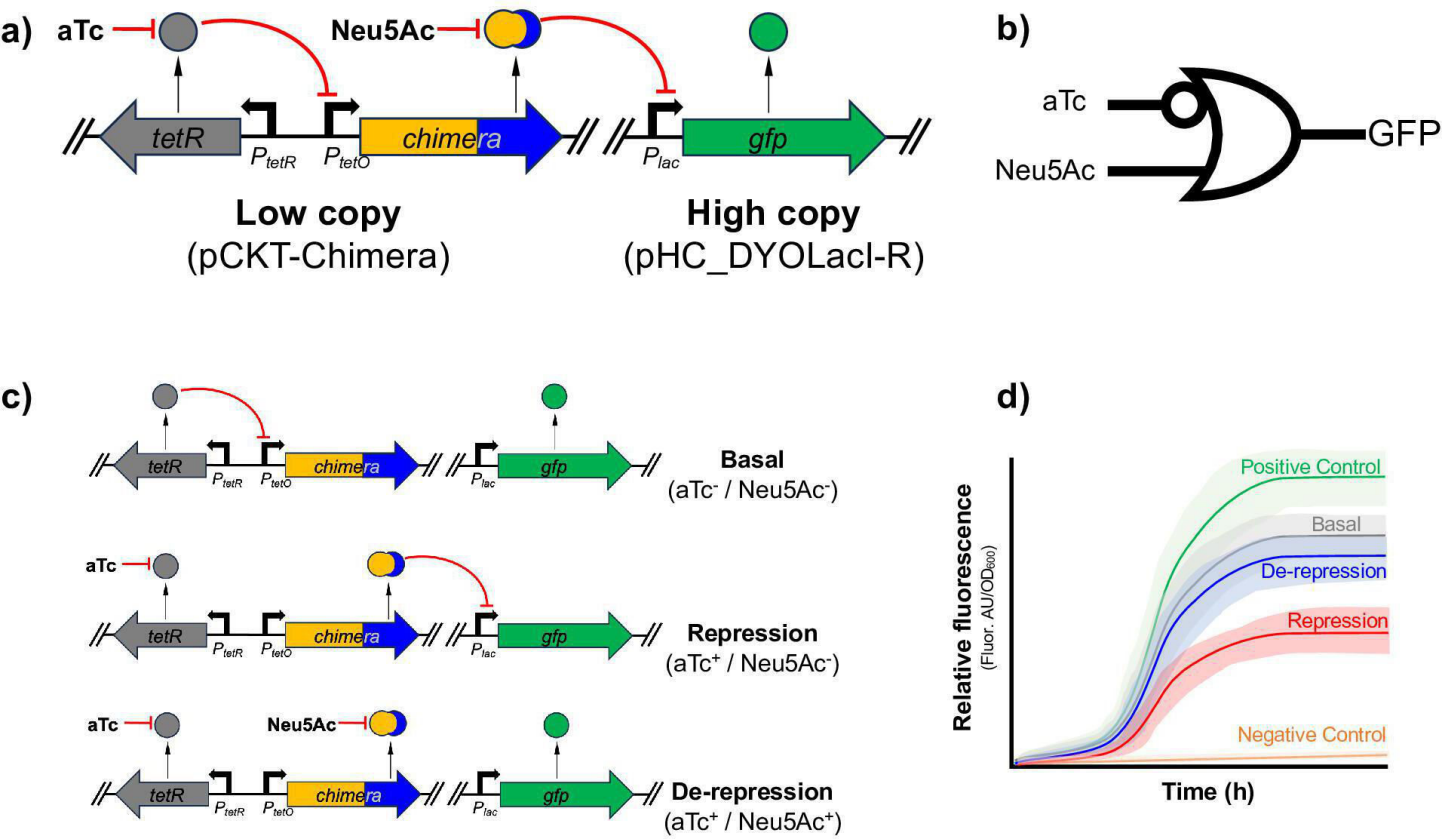
Screening strategy to find functional chimeric transcription factors. (**a**) Genetic circuit to test *in vivo* the functionality of cloned chimeric TFs. MG1655 (pCKT-*Chimera*, pHC_DYOLacI-R) cells express a chimeric TF under the control of TetR. The chimeric product can repress in *trans* the expression of a GFP reporter unless Neu5Ac is present. *tetR*/TetR (gray arrow/circle); chimeric TF gene/protein (gold-blue arrow/circle); *gfp*/GFP (green arrow/circle). (**b**) Standard representation of the B-or-not-A universal two-input gate that governs the expression of GFP in the circuit. (**c**) States of the genetic circuit. Basal (aTc^-^), TeR represses chimera expression, and GFP is produced. Repressed (aTc^+^), TetR is induced, thus the chimeric TF is produced, repressing the promoter expressing *gfp.* De-repressed (aTc^+^, Neu5Ac^+^), the chimera is produced and, if functional, gets induced by Neu5Ac, enabling *gfp* expression. (**d**) Expected fluorescence production over time by a bacterial culture expressing the biosensor genetic circuit and a functional chimeric TF. Positive control (green line), corresponding to a strain with a constitutive *gfp.* Negative control (orange line), background fluorescence by a *gfp^-^* strain. Basal (gray line), Repressed (red line), De-repressed (blue line): fluorescence associated with a MG1655 (pCKT-*Chimera*, pHC_DYOLacI-R) culture expressing a chimera in the basal, repressed, and de-repressed states, respectively.

The assessment of WCB functionality through genetic circuits that drive protein expression is a mainstay of bioreporter design and a fertile arena for innovation in detecting small, soluble molecules (75). A functional *in vivo* screening was implemented using GFP as a reporter. Plasmid pHC_DYOLacI-R (Table S2) was transformed into candidate strains expressing the chimeric TFs from pCKTRBS vectors under the control of TetR-aTc. These two plasmids expressed in the same strain constituted a genetic circuit (Fig. 1a) that enabled a controlled expression of the chimera as well as its controlled induction. This circuit behaves as a B-or-not-A universal two-input gate (76) (Fig. 1b), where there are two inputs (A, aTc supplementation; B, Neu5Ac supplementation) and one output (detection of GFP fluorescence). Based on the addition of the inputs, we can define three states in the genetic circuit (Fig. 1c): Basal (aTc^-^/Neu5Ac^-^), no input added, GFP is expressed from its promoter in pHC_DYOLacI-R; Repression (aTc^+^/Neu5Ac^-^), the presence of aTc induces TetR, which is incapable of remaining attached to *P_tetO_* and thus the chimera is expressed from its corresponding pCKT-*Chimera* vector repressing the expression of GFP; De-repression (aTc^+^/Neu5Ac^+^), the chimera is expressed, but if it is functional, it is then inducible by Neu5Ac, de-repressing the promoter driving the expression of GFP. By comparing the behavior of the strain expressing the aforementioned circuit under these conditions, we can rule out non-functional synthetic TFs. GFP-associated fluorescence data displayed in this publication are always expressed as relative fluorescence (Rel.fluor = fluorescence arbitrary units/OD_600_ × 1,000), which includes a correction of fluorescence by the optical density of the culture to account for growth disparities among different strains and conditions. In the case that the chimeric protein was not a functional repressor, relative GFP-associated fluorescence (Rel.fluor) could be detected in the Repression state (ΔRel.fluor_basal-repressed_ tending to 0). On the other hand, if the chimera was able to bind to *lacO* on the reporter promoter yet incapable of recognizing Neu5Ac with its LBD, or to transmit a LBD to DBD conformational change resulting in its detachment from the operator, we would have obtained a functional super-repressor (77) unable to respond to its inducer and, thus, not a valid tool to detect Neu5Ac [ΔRel.fluor_(de-repressed)-repressed_ tending to 0]. Figure 1d illustrates the expected GFP production along a growth curve for a strain expressing the screening genetic circuit that included an ideal chimera [ΔRel.fluor_(de-repressed)-repressed_ >0]. It is important to note that even though Neu5Ac is a monosaccharide, its supplementation to rich growth media did not significantly increase the OD_600_ of the assayed strains, at least up to the tested 10 mM concentration (Fig. S1). It is also important to note that repression by LacI, or LacI-derived chimeras, is rarely absolute, so that a certain level of leakage, and thus of background reporter gene expression, is always expected (21, 78).

All the chimeric TFs included in Table 1 were screened for their ability to bind DNA and thus to repress the expression of the GFP reporter, as well as to be induced by Neu5Ac, enabling production of the fluorescent protein. All members of the collection of MG1655 (pCKT-*Chimera*, pHC_DYOLacI-R) strains bearing the genetic circuit described in Fig. 1 were tested *in vivo* to assess their ability to behave as Neu5Ac biosensors (Table S4). Among the chimeric proteins expressed by this strain collection, we included the TF Lumos (LacI-LNK6-SiaP), which did not contain any of the expected LNK1-5 options but an unexpected six amino acid linker (LNK6) resulting from an abnormal assembly reaction in which the last 18 bases of *DBD-lacI* were duplicated (Table S1). Given the stable nature of the amino acid string, as well as our agnostic approach to which LNK sequence could be the best performing one, we decided to maintain this construction in our pool. TFs Kunst (LacI-LNK3-SiaP), Scorpio (LacI-LNK4-SiaP), Lumos (LacI-LNK6-SiaP), and Haus (LacI-LNK2-SatA) showed no repression abilities. We can only hypothesize about the causes for the lack of observed repression in the aforementioned chimeras. Two potential explanations would be an inability to interact allosterically to create a stable dimer capable of binding to *lacO*, or the possibility that the chimeras are misfolded and thus inactive and/or even toxic. The latter would be congruent with the diminished growth presented by the strain bearing Haus, which suggests the potential deleterious effect of this protein for the host cell.

Bastet (LacI-LNK5-SiaP) was intriguing due to the possibility that it was able to repress and de-repress for the first 10 h of growth, yet its behavior requires a deeper study given the observed dispersion in the analyzed data. This observation suggests that the strain might be unstable due to the potential toxicity of the protein. Also of note, Harpy (LacI-LNK1-SatA) seemed to be able to repress at a certain level and potentially de-repress after the 18-h mark. Another TF that presented a similar and unexpected induction profile but on a more consistent basis was Siren (LacI-LNK1-SiaP), which displayed an intriguing function as a proper repressor inducible by Neu5Ac but only in the stationary phase of growth (Fig. S2). Our data suggest that this late induction is not due to an increased metabolic rate caused by an eventual consumption of Neu5Ac as a carbon source in the stationary phase, since, as can be observed in Fig. S1, supplementation of growth media with Neu5Ac does not alter growth at any growth stage. In the future, we intend to study this protein in depth, since its repression/de-repression profile is very attractive for a WCB setup in which a bacterial culture sustaining itself at the stationary phase is continuously monitoring an input for an extended period of time. Prolonging the functional time of WCBs is a known challenge associated with these technologies, and engineering of genetic circuits incorporating regulators such as Siren is one of the strategies that are being explored to account for this problem (4). A candidate biosensor strain expressing Sphnx (LacI-LNK2-SiaP) exhibited the most suitable repression and induction profile compatible with that of a TF capable of repressing *P_lac_* while de-repressing it in a detectable manner upon binding to Neu5Ac (Table S4). It is worth noting that, among the different candidates, SatA-based chimeras seemed to be more difficult to build, and when assembled, they presented worse results than SiaP-based chimeras. This could be owed to an increased toxicity potentially associated with a misfolding of the fusion gene, yet further studies and the creation of expanded chimeric libraries, however, are required to confirm this hypothesis.

**Fig 2:**
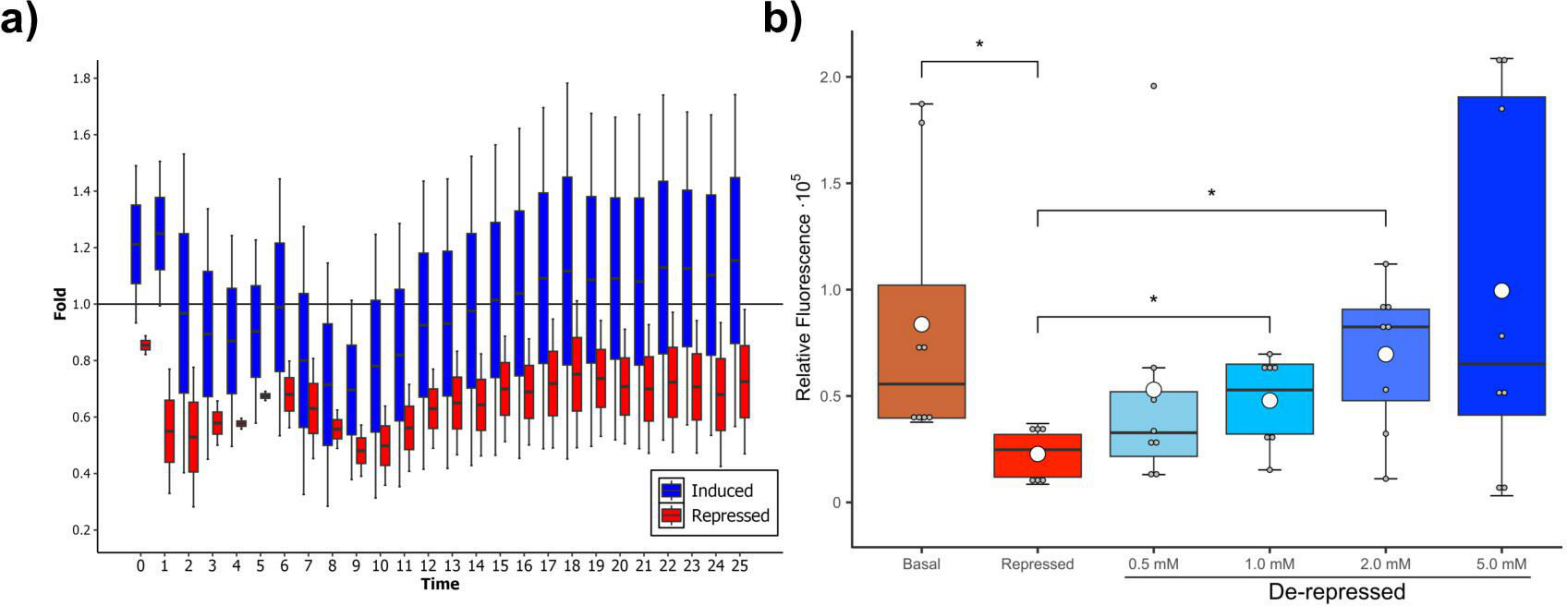
*In vivo* validation of the functionality of Sphnx (LacI-LNK2-SiaP). (**a**) Time course showing relative fluorescence of MG-Sphnx. Promoter activity is measured as fold change of the Rel.fluor, relative fluorescence of the strain growing in the de-repressed (aTc^+^, Neu5Ac^+^; blue) or repressed (aTc^+^, Neu5Ac^-^; red) states compared to a basal expression (aTc^-^, Neu5Ac^-^). Boxplots with whiskers represent data dispersion of the average values of biological replicates (*n* = 8–10). (**b**) Relative fluorescence of MG-Sphnx cultures after induction with different concentrations of Neu5Ac and growth for 14 h. Promoter activity is measured as Rel.fluor of the strain growing in the basal (brown), repressed (red), or de-repressed (blue shades; Neu5Ac concentration in mM) conditions. Boxplots with whiskers represent data dispersion of the average values of biological replicas (*n* = 8; average of the means, white circle). Statistically significant differences between selected conditions are marked with stars (* *P*-value < 0.05).

Looking forward to validating our preliminary observations for an Sphnx-based WCB, we systematically compared GFP production over time of MG1655 (pCKT-Sphnx, pHC_DYOLacI-R), MG-Sphnx for short, cells expressing Sphnx either in the presence or absence of Neu5Ac. Figure 2a displays the results of time course experiments over a period of 25 h. GFP production induced by the presence of Neu5Ac was observed from the first hour of growth, with the Neu5Ac-induced cultures showing a tendency to display higher fluorescence than the repressed ones. Figure S3 shows the behavior of Kunst, which only differs from Sphnx in its LNK and which exhibited a tendency to repress *P_lac_* at the very beginning of a 40 h time course, yet after the 3 h mark did not show any difference between induced and uninduced conditions. Given the similarity between Sphnx and Kunst, and the lack of functionality of the latter, we used it as our reference non-functional chimera. A preliminary evaluation of the structural differences between Sphnx and Kunst is included in its own section of Results and Discussion.

In an effort to validate the *in vivo* observations confirming Sphnx functionality, we analyzed the induction of *P_lac_* driving the expression of GFP in MG-Sphnx by qRT-PCR experiments (Fig. S4*)* performed as indicated in Materials and Methods. The expression levels of *sfgfp* calculated as the ratio of the induced condition over the repressed condition were 1.58 ± 0.24 (*n* = 8, error expressed as SEM), statistically significant and consistent with an induction of the expression in the presence of Neu5Ac.

Further *in vitro* experiments were also performed to confirm the interaction between *P_lac_* and the DBD-LacI of Sphnx. Electrophoretic mobility shift assays (EMSA; Materials and Methods) showed that cell extracts from NEB5-alpha (pCKT-Sphnx) grown for 5 h were capable of retarding the migration of a *P_lac_-sfgfp* probe compared to control extracts that were not expressing Sphnx (data not shown). This indicates that the Sphnx protein expressed from pCKT-Sphnx can bind its cognate promoter *P_lac_*. Preliminary data suggested that Neu5Ac behaved as the inducer of Sphnx, since binding of Sphnx to the probe was diminished in the presence of Neu5Ac in a concentration-dependent manner (Fig. S5). This observation would be owed to the fact that Sphnx is a LacI-derived TF that would behave allosterically: the binding of Neu5Ac to a Sphnx dimer would disrupt its oligomeric state and prevent binding to the *lacO* present in the *P_lac_-sfgfp* probe. On the other hand, cell extracts from NEB5-alpha (pCKT-Siren) grown for 5 h were able to bind the probe but not to unbind in the presence of Neu5Ac, confirming our *in vivo* observations. Previous publications report a similar behavior: DBDs from bacterial transcriptional repressors tend to retain their ability to bind to their operator sequences even when integrated in a new chimeric TF (79–81), another testimony to the high modularity of these proteins. Our initial *in vitro* DNA-protein interaction studies require future validation with purified chimeras to calculate the kinetics of the Sphnx-*lacO* and Sphnx-Neu5Ac-*lacO* interactions, yet they represent a promising step forward toward characterizing the interaction between our synthetic TF and its cognate operator.

At this point, both *in vivo* and *in vitro* experiments strongly suggested that Sphnx was a transcriptional repressor capable of interacting with *lacO* and being de-repressed by neuraminic acid. To better understand the dynamics of the Sphnx-Neu5Ac interaction, we analyzed the production of GFP by MG-Sphnx cultures (Materials and Methods) when exposed to different concentrations of neuraminic acid around the average salivary concentration for an adult male (≈1 mM) (82). The mean fold difference of GFP expression between the repressed and induced conditions was 2.25 ± 0.74 for cultures induced with 0.5 mM Neu5Ac, 2.54 ± 0.52 for cultures induced with 1.0 mM Neu5Ac, 3.81 ± 1.15 for cultures induced with 2.0 mM Neu5Ac, and 5.21 ± 2.00 for cultures induced with 5.0 mM Neu5Ac (*n* = 8, error expressed as SEM). These results were commensurable with those observed for modular chimeric TFs in the presence of their ligands: SLCP_GL_ (LacI-GGBP-OD) induced by glucose (21), CbnR-ABE44898-OD/ChTFBz01 and LmrR-BzdB1_nSP induced by benzoate (18). Figure 2b shows the levels of GFP fluorescence observed in basal and repressed cultures, as well as in cultures induced by 0.5, 1.0, 2.0, and 5.0 mM neuraminic acid. A one-way Welch’s ANOVA was conducted to compare the effect of different Neu5Ac concentrations on Rel.fluor (*F* = 5.3, *df* = 5, *P*-value < 0.005), followed by subsequent t-student pairwise comparisons. These assays corroborated that relative fluorescence between basal (aTc^-^/Neu5Ac^-^) and repressed (aTc^+^/Neu5Ac^-^) conditions was significant. Cultures in which the genetic circuit used to test Sphnx were de-repressed (aTc^+^/Neu5Ac^+^) displayed statistically significant values (*P*-value < 0.05) compared to the repressed cultures when in the presence of 1.0 and 2.0 mM Neu5Ac. Induction by 0.5 and 5.0 mM was not significant. It is important to note that relative fluorescence values in the 5.0 mM-induced biological replicates were widely dispersed. Previous studies have demonstrated that elevated intracellular concentrations of Neu5Ac are toxic for *E. coli* K12 strains (83), suggesting that an overabundance of Neu5Ac, even though it does not seem to alter *E. coli* growth in the assayed conditions, might have other metabolic effects that may affect protein expression down the line, affecting the synthesis of Sphnx and GFP, which in practical terms means that using these biosensors for out-of-the-lab applications may entail the testing of several sample dilutions to ensure proper Neu5Ac detection.

**Fig 3:**
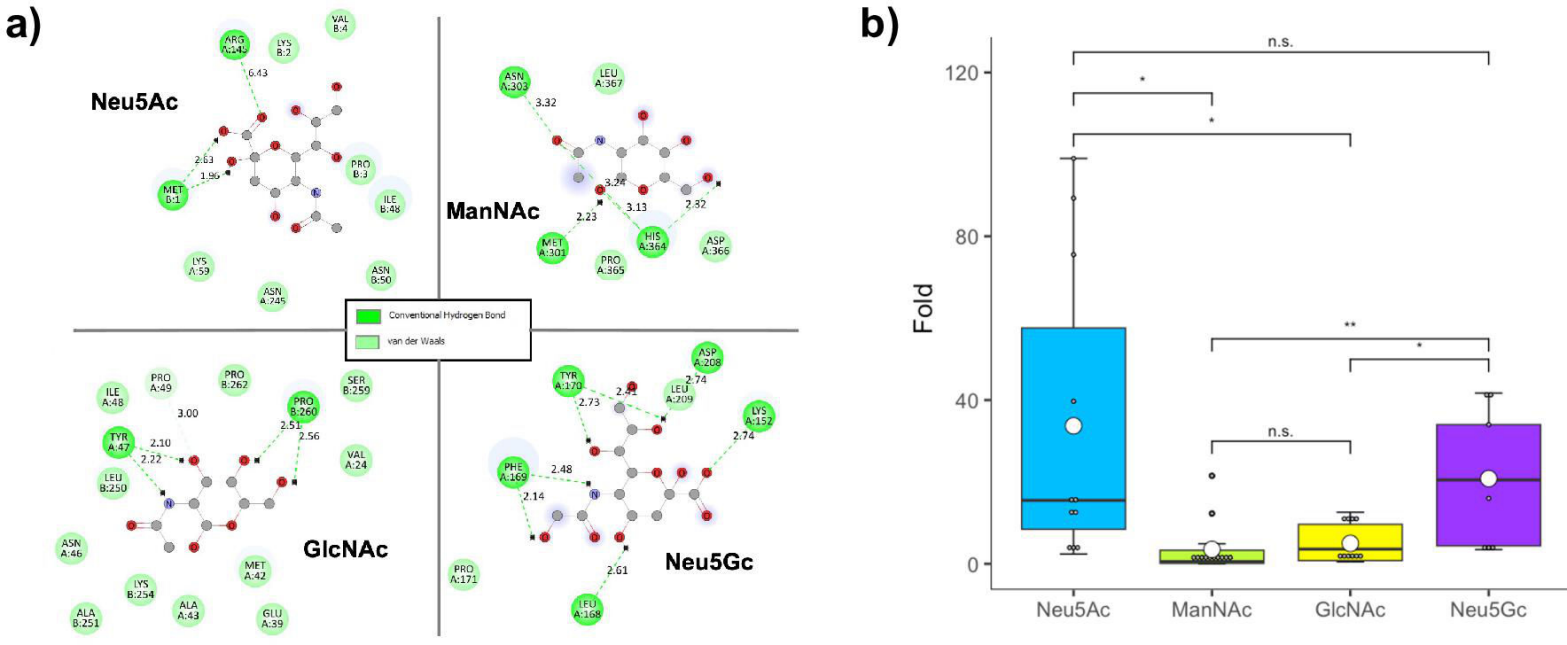
Interaction of Sphnx (LacI-LNK2-SiaP) with different ligands. Analysis of the interaction of Sphnx with N-acetyl-D-neuraminic acid (Neu5Ac), N-acetyl-D-mannosamine (ManNAc), N-acetyl-D-glucosamine (GlcNAc), and N-glycolyl-neuraminic acid (Neu5Gc). (**a**) Molecular docking simulations for Sphnx and ligand molecules displaying stabilizing van der Waals forces and hydrogen bonds between the binding pocket of the regulator and the ligands. (**b**) Relative fluorescence of MG-Sphnx cultures after induction with 1 mM Neu5Ac (blue), ManNAc (green), GlcNAc (yellow), or Neu5Gc (purple). Promoter activity measured as fold change of Rel.fluor over a culture in the repressed condition (aTc^+^, ligand^-^). Boxplots with whiskers represent data dispersion of the average values of biological replicates (*n* = 9–12; average of the means, white circle). Statistically significant differences between selected conditions are marked with stars (*P-value < 0.05; ** *P*-value < 0.01; n.s. non-significant).

To determine the specificity of Sphnx for Neu5Ac, we analyzed the production of GFP by MG-Sphnx cultures exposed to 1 mM Neu5Ac compared to cultures supplemented with 1 mM of either of the following analog compounds: N-acetyl-D-mannosamine (ManNAc), N-acetyl-D-glucosamine (GlcNAc), or N-glycolyl-neuraminic acid (Neu5Gc) (Materials and Methods). ManNAc and GlcNAc are aminated monosaccharides that are part of the biosynthetic pathway of Neu5Ac, constituting the backbone of the molecule (84–86). Neu5Gc is a sialic acid that only differs from Neu5Ac in a single hydroxyl group and, interestingly, cannot be synthesized by humans, originating from diet and microbiota production (87–89). Neu5Gc has been postulated as a cancer biomarker due to its proinflammatory effects (90, 91). Molecular docking simulations (Fig. 3a) strongly suggested that both Neu5Ac and Neu5Gc could theoretically establish more stabilizing bonds with the amino acids present in the binding pocket of Sphnx-LBD, whereas ManNAc and GlcNAc would be able to enter said pocket, yet their interaction with it would be extremely weak. These observations were corroborated *in vivo.* Figure 3b displays GFP-fluorescence levels by MG-Sphnx cultures exposed to the different Neu5Ac analogs. Induction by the different compounds is expressed as fold change Rel.fluor (*n* = 9–12, error expressed as SEM) of each induced culture (aTc^+^/ligand^+^) over a reference repressed condition (aTc^+^/ligand^-^). A one-way Welch’s ANOVA was conducted to compare the effect of different ligands on Rel.fluor (*F* = 15.8, *df* = 4, *P*-value < 0.005), followed by subsequent t-student pairwise comparisons. No statistically significant difference was found between repressed and ManAc^+^ or GlcNAc^+^ conditions, strongly suggesting that these compounds are not structurally similar enough for Sphnx to bind them. However, Neu5Gc^+^ cultures displayed a significant induction compared to the reference (*P*-value < 0.05), presenting on average 62% ± 15% of the Neu5Ac^+^ induction. Upon further analysis, there was no statistically significant difference between Neu5Ac^+^ and Neu5Gc^+^ datasets. These findings confirm that Neu5Gc is an inducer of Sphnx, which, upon further review of the existing literature, was consistent with previous observations that described how SiaP (Sphnx-LBD) was able to bind Neu5Gc *in vitro* with μM affinity (70) simulating human saliva (Materials and Methods). It has also been described that Neu5Ac transporters can also import other sialic acids, such as Neu5Gc (92), which could contribute to the enhanced detection of this metabolite. Neu5Gc is not synthesized by human cells but is instead acquired through the diet (88, 93, 94) and preferentially accumulates in the tissues of several tumors (95), leading to its proposal as an inflammation and cancer biomarker in its own right (96). An electrochemical biosensor, employing an aptamer conjugated to boronic acid, enables highly specific and efficient detection of Neu5Gc (97).

MG-Sphnx cultures mixed with stabilized mucin-containing artificial saliva supplemented with Neu5Ac for a final 1 mM concentration returned a Rel.fluor signal 3.2 ± 0.9 fold (mean ± SEM, *n* = 5) higher than samples of unsupplemented saliva. These data, even though preliminary, are very promising for the capabilities of WCBs for diagnostic applications using clinical samples. Even though Neu5Ac has been postulated as a biomarker for oral cancer and dysbiosis (98, 99), there is no standard analysis for its quantification. Colorimetric assays based on thiobarbituric acid (Warren assay) (48) have been refined over time for their use on salivary samples (100) and, more recently, adapted for their use with gold nanoparticles (101). The latter is a good representative of the capabilities of colorimetric methods, with a limit of detection of 0.06 mM and exhibiting linearity between 0.15 and 1.00 mM. A major advantage of these detection protocols over WCB-based approaches is their reduced assay time, typically around 5 min. Following a different approach, enzymatic (102), amperometric (103), and chromatographic (e.g., liquid chromatography plus tandem mass spectrometry, LC-MS/MS (104) assays have been proposed. The latter LC-MS/MS application provides detection limits of 3.69 nM. Alternatively, an antibody-based indirect competitive ELISA (ic-ELISA) (105) and a chemiluminescence enzyme-linked immunosorbent assay (CLEIA) (50) detect Neu5Ac in a similar range that LC-MS/MS (1.84 and 0.88 nM, respectively) while requiring a simpler sample preparation. These methods are generally more sensitive than any WCB, including MG-Sphnx, but they require more sophisticated equipment and trained personnel. If we compare the performance of our biosensor strain with other WCBs, we can observe that MG-Sphnx operates in a similar range to other Neu5Ac biosensor strains. Different Neu5Ac-sensing circuits have been deployed in strains capable of synthesizing Neu5Ac. Biosensors based on the native *E. coli* NanR have detection limits ranging from 0.3 mM (52, 106) to 4.5 ± 1.3 mM (107). In heterologous hosts, *E. coli* NanR-based sensors can detect 0.3 mM of exogenous Neu5Ac when expressed in *B. subtilis* (106) and display linearity between 1.0 and 5.0 mM in *Synechococcus* spp. (108). The RipR-family NanR from *Clostridium perfringens* displayed a detection limit of 30 mM Neu5Ac (exogenous) (57). The fact that MG-Sphnx can report exogenous Neu5Ac concentrations so similar to those reported by native regulators is very promising. We want to note the potential for improvement of the chimeric protein, once we advance on our study of its behavior *in vivo* and *in vitro*, based on known strategies such as enhancement of domain interaction (via rational mutagenesis), refinement of induction range, as well as gene dose and reporter gene optimization (17). Newly created synthetic TFs tend to have limited transfer function and specificity, and thus to require further engineering for their field deployment in biosensors (109). In this regard, we are optimistic about the future improvement of the chimeras we have assembled: the estimated *K_d_* of SiaP and SatA for neuraminic acid are in the nM range (67, 68, 70) (calculated *in vitro*), whereas in this work, we present data in the mM range (calculated *in vivo*). This suggests that we have not reached the full potential of our LBDs, and there is an opportunity to optimize Sphnx’s structure to better benefit from the high specificity and affinity of our LBD. It is also important to note that Sphnx is currently at the same point in evolutionary history when DBD-LacI was fused to the ancestral version of its current LBD, long before communication between DBD and LBD had been refined and perfected by natural selection (61). A systematic exploration of promoter and RBS variants can provide a better solution to compensate for some of these deficiencies (17). The use of synthetic unregulated weak promoters (e.g., [110]) for the expression of the chimera rather than its controlled expression by TetR/aTc might contribute to easing some of the observed anomalies in the expression of the fusion genes created in this project. An interesting avenue of research would be the generation of chimeric TFs using, as LBDs, proteins capable of binding Neu5Ac yet entirely unrelated to SiaP and SatA (111). Regarding the use of alternative reporters that may shed more light on the induction profile of Sphnx, we acknowledge that the use of GFP has some inherent limitations in prokaryotes, such as widely varying maturation times (112, 113) dependent on strain and metabolic state (112). This is a strong motivation to explore alternative fluorescent reporters with shorter half-lives (e.g., mJuniper [114]) in future iterations of MG-Sphnx as well as to initiate the development of novel alternative reporting systems. Finally, biosensor strains such as MG-Sphnx can benefit from the modulated expression of transporter genes, increasing the uptake of the analyte into the strain. Our MG1655 biosensor chassis carries a native Neu5Ac importer, NanT (83, 115), whose expression can be fine-tuned in the future to detect different levels of our target biomarker. It would be interesting to compare the sensitivity of MG-Sphnx expressing NanT to a new Neu5Ac biosensor based on a fusion of SiaP to EnvZ from the two-component signaling system EnvZ-OmpR (23). In this case, SiaP would become the periplasmic, yet tethered rather than free, module of EnvZ, directly interacting with Neu5Ac without the need to import it into the cytoplasm.

Beyond any discussion on the sensitivity of MG-Sphnx, it is important to note that our Neu5Ac confronts an issue common to most WCBs: dilated detection times extending over many hours, reducing their immediate practical use (116, 117). To test the feasibility of shorter Neu5Ac detection assays, we grew MG-Sphnx cells, washed them to remove rich medium, concentrated them in minimal medium lacking a carbon source, and then exposed them to 1 mM Neu5Ac (Fig. S6). Under these conditions, where WCB concentration has been incremented at time 0, and growth was limited by the absence of a carbon source, we observed statistically significant differences in Rel.fluor (*n* = 8, *t*-test, *P*-value < 0.01) between bacterial suspensions exposed to Neu5Ac and control samples after 4 h of incubation. This assay time is on par with other comparable GFP-based WCBs such as the quorum-sensing-based detector for waterborne bacterial pathogens designed by Wu et al. (5). On average, Neu5Ac-induced cultures presented 1.22 ± 0.04 fold (mean ± SEM, *n* = 8) higher Rel.fluor than uninduced cultures. These preliminary results are promising and suggest that detection times can be partially reduced by adjusting the initial cell concentration, a strategy we plan to investigate in depth in the future. Another alternative approach that will contribute to reducing the assay times is the use of reporters other than fluorescent proteins. For example, the strain built by Barger et al. to detect blood markers in urine is a TF-based (HrtR from *Lactococcus lactis*) WCB, expressing a transporter to improve sensitivity (ChuA from *E. coli*), that is able to detect heme in the 1–2 h time frame by using bioluminescence (*lux* system) as a reporter (118). Assessing the luminescence produced by the heme-exposed culture with an optoelectronic sensor streamlines the assay and makes it more practical to use. One avenue we are exploring is the engineering of key regulatory networks in the *E. coli* MG1655 chassis to enhance its electrogenic capabilities, generating an MG-Sphnx variant capable of reporting the presence of Neu5Ac by directly producing an electronophore-mediated electrical current, hence reducing signal attenuation caused by intermediate relay steps. Atkinson et al. established an orthogonal strategy by coupling thiosulfate detection to electricity generation in a strain expressing a synthetic electron transfer chain for direct electron transfer, rather than relying on shuttle molecules, contributing to demonstrate the feasibility of bioelectricity as a rapid-response reporter in an *E. coli* chassis (119).

### Modeling of the 3D structure of chimeric transcription factors

**Fig 4:**
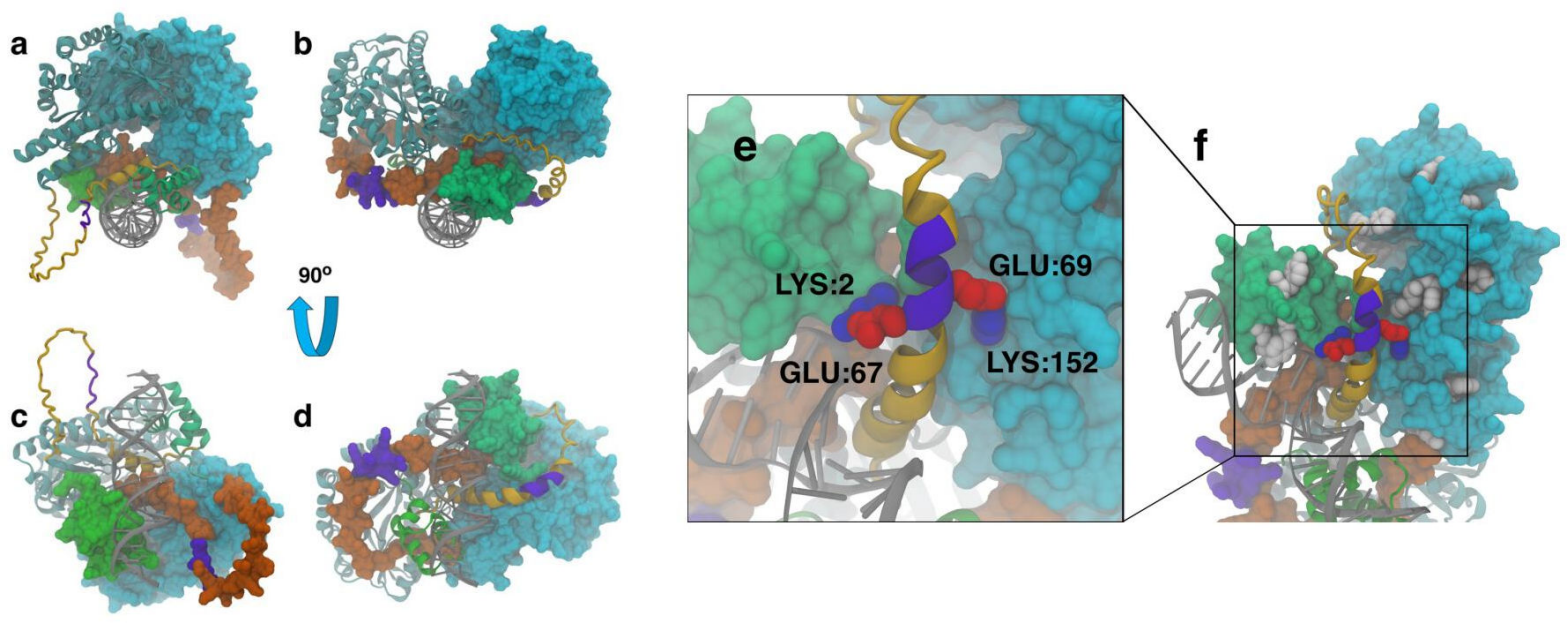
Modeled structures of chimeric protein dimers with double helix DNA obtained with Alphafold 3. For each structure, one monomer is shown using the New Cartoon representation and the other using the space-filling Surf representation. The DBD is shown in green, the PBP in cyan, and the connecting chain in orange, with the engineered linker in violet. The Kunst (**a**) and Sphnx (**b**) dimers are shown looking down the axis of the double helix, along with the rotated Kunst (**c**) and Sphnx (**d**) structures. The next panels contain the zoomed-in helical protein (**e**) linker (violet) in contact with the DBD (green) and PBP through the formation of salt bridges, with acidic residues shown in red and basic residues in blue. The zoomed-out protein (**f**) highlights the location of aromatic residues (white) that can be used to explore the validity of predicted structures using fluorescence anisotropy.

Another element we are starting to explore is the importance of linkers for the functionality of modular proteins, an often-neglected design element (120). Since the beginning of our journey characterizing the chimeric TFs, we found extremely intriguing the fact that Sphnx (LacI-LNK2-SiaP, N_t_-DBD-EKEKEK-LBD-C_t_) and Kunst (LacI-LNK3-SiaP, N_t_-DBD-GSGSGS-LBD-C_t_), which are nearly identical proteins (see relevant transcription factor sequences in the supplemental material), possessed opposed behaviors when interacting with neuraminic acid. The recent release of the AlphaFold 3 software (121) has enabled us to predict the structures of multimeric chimeric proteins in the presence of their dsDNA operator, allowing us to model the proteins under conditions closer to those found in actual WCBs. It is important to note that the structures predicted by AlphaFold 3 may not be the actual structures occurring in the cell, or may be part of an ensemble of many 3D configurations that occur *in vivo*; however, the predicted structures are still based on reasonable biophysical interactions and can provide insight into the types of interactions one would expect to observe. In this way, Alphafold 3 can be used as a hypothesis-generating tool for understanding the protein-protein interactions involved in the function of chimeric proteins. Sphnx and Kunst were both modeled as dimers using AlphaFold 3, along with a DNA double helix containing the *lacO* (5′-ATTGTGAGCGGATAACAATT-3′ and its complementary sequence) recognized by the DBD-LacI they both carry. Our goal was to predict structures that suggest plausible and reasonable interactions that could help explain why Sphnx binds Neu5Ac while Kunst does not, despite only differing by six amino acids. Figure 4a shows the predicted Kunst DBD (green) bound to the DNA double helix (gray) and the formation of a dimer between the PBP (cyan). The predicted structure shows a mostly disordered linking region (orange), including the GSGSGS (LNK3) linker (violet). The predicted Sphnx model (Fig. 4b), on the other hand, shows that the connecting region adopts a helical structure, including the EKEKEK (LNK2) linker, and appears to form contacts between both the DBD and PBP. Rotating the Kunst structure by 90° (Fig. 4c), we can observe that the GSGSGS (linker) does not interact with the protein or DNA. In contrast, rotating the Sphnx structure by 90° (Fig. 4d) further shows that the linker forms contacts between the DBD and PBP domains, with interactions occurring across different monomer chains. Further analysis shows that the predicted helical linker in Sphnx forms electrostatic salt bridges between itself and both the DBD and PBP domains. Figure 4e shows the acidic residues on the linker (Glu) forming contacts with basic residues (Lys) on the DBD and PBP domains. Specifically, the predicted structure suggests that the residues expected to form salt bridges include Glu67-Lys2 on the DBD and Glu69-Lys152 on the PBP. These salt bridges likely stabilize the helical linker, affecting the flexibility and stability of the polypeptide, an effect that has been previously observed in other proteins (122). From these predicted structures, we hypothesize that, in general, these salt bridges may act to stabilize helicity in the flexible linker in a way that positively affects the PBP function by interacting with the DBD and the PBP, explaining the ligand binding functionality of Sphnx compared to the non-binding Kunst protein. Because of the E and K amino acids on the Sphnx linker, these electrostatic interactions may not be specific to the residues we observed but may act as a general way to stabilize interactions between the domains and the linker. Zooming out to see the larger structure (Fig. 4f), aromatic residues near the linker salt bridges are visible. These residues are key for testing the new predicted structures and designing new chimeric proteins in the future. We intend to purify Sphnx and Kunst to perform fluorescence analysis of the protein-ligand interactions, including fluorescence quenching and fluorescence anisotropy studies. The construction of 3D models, using the latest version of AlphaFold, will enable us to explore which mutations might enhance ligand binding in a way that was previously unavailable, also allowing us to model conditions closer to the actual multimeric *in vivo* structures.

### Conclusions

This work presents MG1655 (pCKT-Sphnx, pHC_DYOLacI-R), MG-Sphnx, the first iteration of a transcription factor-based whole-cell biosensor strain capable of detecting the salivary biomarker N-acetyl-D-neuraminic acid (Neu5Ac) and reporting its presence through the production of a fluorescent reporter. This target molecule has been postulated as an attractive analyte to monitor since its elevated levels in saliva are correlated with conditions that range from periodontal disease (82) to the onset of oral squamous cell carcinoma (40–42). MG-Sphnx's ability to detect N-glycolyl-D-neuraminic acid (Neu5Gc) as well expands its potential use as a biosensor for the detection of overall sialic acid imbalances associated with multiple dysbiotic, inflammatory, and neoplastic conditions (53, 91, 123, 124). This study contributes to the literature on the construction of affordable custom-made TFs in a field limited by the availability of sensing modules (17, 61). The modular assembly of fusion genes such as Sphnx sheds light on the importance of inter-domain communication for multi-domain protein functionality (125) and exemplifies how a reduced number of independent modules can synergistically originate new functionalities in the cell (126). It is important to note that synthetic TFs such as Sphnx do not represent an endpoint, but the origin of a new source of regulators whose performance can be improved (127–129). We have obtained promising results on the functionality of MG-Sphnx to detect N-acetyl-D-neuraminic in preparations of artificial saliva (130); however, further experiments are needed to improve the assay to detect a wider range of biomarker concentrations.

## MATERIALS AND METHODS

### Bacterial strains and growth conditions

Bacterial strains and plasmids used are listed in Table S2*. E. coli* strains were grown at 37°C in lysogeny broth (LB [131]) medium unless otherwise indicated. When required, *E. coli* cells were grown in M63 minimal medium (132) using the necessary nutritional supplements and 30 mM glycerol (Sigma, St. Louis, MO) as a carbon source. Antibiotics were added at the following concentrations: 100 μg·mL^−1^ ampicillin/carbenicillin and 25 μg·mL^−1^ chloramphenicol (Sigma, St. Louis, MO). For protein expression from pCKTRBS-derived vectors, cultures were induced with 0.5 μg·mL^−1^ anhydrotetracycline (aTc) (Clontech, Mountain View, CA). When experimental conditions required it, cultures were supplemented with N-acetyl-D-neuraminic acid (neuraminic acid, Neu5Ac; Sigma, St. Louis, MO), N-acetyl-D-mannosamine (ManNAc; TCI Chemicals, Portland, OR), N-acetyl-D-glucosamine (GlcNAc; TCI Chemicals, Portland, OR), or N-glycolyl-neuraminic acid (Neu5Gc; AFB Bioscience-VWR, Radnor, PA) for a 1 mM concentration unless otherwise indicated.

Culture growth was routinely monitored in a BioPhotometer D30 (BioSpectrometer Basic Kinetic and Fluorescence; Eppendorf, Hamburg, Germany). When it was required, bacterial growth and GFP production were recorded in a Varioskan LUX Multimode Microplate Reader (Thermo Fisher, Waltham, MA) unless otherwise indicated. Cultures were pipetted on a 96-well plate (Flat bottom Polystyrene Black with clear bottom; Corning, Corning, NY) covered with a Breathe-Easy BEM-1 gas-permeable membrane (Diversified Biotech, Dedham, MA) and incubated in a plate reader, where OD_600_ and fluorescence in the range of GFP (excitation 485 nm, emission 528 nm) were either tracked along time courses (up to 40 h) or subjected to point measurements. NEB5-alpha (pHC_DYOLacI-R) and MG1655 were included in every plate as GFP-positive and GFP-negative reference strains, respectively. For standard time course experiments, overnight precultures of the strains were used to inoculate (starting OD_600_ 0.025) fresh growth media (supplemented with aTc and Neu5Ac when indicated) in 96-well plates (180 μL total volume per well).

### Neu5Ac detection assays with MG-Sphnx

The assay described in Fig. 2b, in which the MG-Sphnx strain is exposed to different concentrations of Neu5Ac, started with 5 mL overnight precultures of the strains (plus aTc and Neu5Ac when required) that were used to inoculate 5 mL of fresh media (1:50) supplemented in the same manner as the precultures. After 14 h of growth, 180 μL samples of the cultures were transferred to 96-well plates to enable OD_600_ and fluorescence readings (single-point measurements).

The assay described in Fig. 3b, in which MG-Sphnx cells are exposed to 1 mM Neu5Ac or 1 mM of analog compounds (ManNAc, GlcNAc, and Neu5Gc), started with 2 mL overnight precultures of the strain (plus aTc and Neu5Ac/ManNAc/GlcNAc/Neu5Gc when required) that were subsequently used to inoculate 2 mL of fresh media (1:50) supplemented as the precultures. After 20 h of growth, 180 μL samples of the cultures were transferred to 96-well plates to enable OD_600_ and fluorescence readings (single-point measurements).

The experiments using artificial saliva began with a 5 mL overnight preculture of MG-Sphnx, supplemented with aTc, that was used to inoculate (with 20 μL) a mixture of 750 μL of fresh media (plus aTc) and 250 μL of artificial saliva (artificial saliva with mucin, stabilized, Ref. 1700-0316; Pickering Laboratories, Mountain View, CA). The mix was incubated for 22 h at 37°C in an orbital shaker to ensure proper mixing and favor strain growth. This prolonged incubation seems to be key for the homogenization of the sample, since initial attempts to grow the mixture of culture and artificial saliva under the gentler shaking conditions provided by the plate reader failed, presumably due to the viscosity of the saliva. Once the incubation was finished, 180 μL samples of the cultures were transferred to 96-well plates to perform OD_600_ and fluorescence readings (endpoint measurements). Incubation of the samples in the plate reader for up to 48 h, after the endpoint measurement, with continuous OD_600_ and fluorescence monitoring did not provide more informative data.

The proof-of-concept faster assays described in Fig. S6, in which the ability of MG-Sphnx to detect Neu5Ac under marginal growth conditions is tested, was prepared as follows. Three milliliters of MG-Sphnx overnight cultures in LB supplemented with aTc was pelleted, and the cells were washed twice with PBS (pH 7.2). The final pellet was resuspended in 1 mL of M63 media supplemented with aTc, chloramphenicol, and carbenicillin but lacking any carbon source (M63_aTc-Cm-Ap_). For every detection reaction, 500 μL of these preparations was mixed with 500 μL of either _M63aTc-Cm-Ap_ or M63_aTc-Cm-Ap_ supplemented with Neu5Ac for a final well concentration of 1 mM. 180 μL of samples was transferred to a 96-well plate that was incubated at 37°C without shaking. After 4 h, OD_600_ and GFP-associated fluorescence readings were taken in a Spark Multimode Microplate Reader (TECAN, Männedorf, Switzerland).

### Molecular biology techniques

Molecular biology techniques were performed following commonly used standard protocols and as per the manufacturers’ instructions (133). PCRs took place in a 6321 Mastercycler PRO Vapo Protect Thermal Cycler (Eppendorf, Hamburg, Germany). Routine separation of DNA and RNA in agarose gels was performed in a RunOne Electrophoresis system (EmbiTec, San Diego, CA). Agarose gels were imaged in a UVP UVSolo Touch (Analytic Jena, Jena, Germany). Plasmid DNA was purified with a Qiaprep Spin Miniprep Kit (Qiagen, Hilden, Germany). DNA fragments were purified with the DNA Clean-up and Concentration Kit (Zymo Research, Irvine, CA). The oligonucleotides employed for PCR amplification of the cloned fragments and other molecular biology techniques are summarized in Table S3 and were supplied by IDT (Coralville, IA). All cloned inserts and DNA fragments were confirmed either via Sanger sequencing (134) performed by Azenta Inc. (Burlington, MA) or Oxford-Nanopore whole-plasmid sequencing performed by Plasmidsaurus (Eugene, OR). Commercially available NEB5-alpha chemically competent cells (NEB, Ipswich, MA) were used for routine transformations. Alternatively, electrocompetent *E. coli* cells were generated and transformed immediately (Gene Pulser; Bio-Rad, Hercules, CA) (133). Nucleotide sequence analyses were done at the National Center for Biotechnology Information server (https://www.ncbi.nlm.nih.gov/) and Benchling Biology Software (https://www.benchling.com/). Cloning was routinely performed via Gibson assembly (135) unless otherwise indicated.

### Domain selection and cloning

DBD-LacI is located on *lacI* 5′ region, from the start codon to base 198, encoding a peptide of 66 amino acids that comprises the N-terminus of our chimeric proteins. This domain was obtained via PCR amplification from plasmid pCKTRBS-LacIwt (18) using the oligonucleotide pair SV00001/SV00002. SatA from *H. ducreyi* (Uniprot Q7VL18) and SiaP from *H. influenzae* (Uniprot P44542) were ordered as synthetic dsDNA fragments (IDT, Coralville, IA). Several variants of the LBDs SatA and SiaP were amplified via PCR so that they would include 5′ overhangs, including linkers (LNK2-5) or no linker whatsoever (LNK1). SatA was amplified with primers SV00030-SV00034/SV00004 and SiaP with primers SV00015-SV00019/JFJ0018.

Genes encoding the chimeric TFs were originally assembled in pUC19 (136). The vector was amplified via divergent PCR with primers JFJ00019/JFJ00020, which introduce a consensus RBS for *E. coli*, originating from plasmid pUC19RBS. Tripartite Gibson assemblies, including linear pUC19RBS, DBD-LacI, and LNK-LBD, were performed, and the resulting constructions were transformed into NEB5-alpha competent cells. After a carbenicillin selection in solid media, a subset of the resulting clones was isolated and their plasmids (pUC19-*Chimera* family, Table S2) purified and sequenced to validate the presence of chimeric TFs. The resulting TF genes cloned into pUC19RBS were amplified using SV00024/SV00025 for SiaP-derived chimeras and SV00024/SV00026 for their SatA counterparts. These primers amplify our TF genes from the 5′ DBD-LacI, just downstream of the consensus RBS site, to the stop codon of the LBD. Low copy plasmid pCKTRBS (18) was amplified via divergent PCR using primer pairs SV00022/SV00021 for SiaP-derived chimeras and SV00022/SV00023 for their SatA counterparts. These primers introduce 5′ and 3′ overhang sequences providing homology to our dsDNA chimeric gene fragments, and including in their 5′ side our consensus RBS site. Bipartite Gibson assemblies, including linear pCKTRBS and the fusion genes encoding the chimeras, were performed, and the resulting assembly products were transformed into NEB5-alpha competent cells. After a carbenicillin selection in solid media, resulting clones were isolated and their plasmids sequenced to confirm the presence of chimera-encoding genes. Resulting pCKT-*Chimera* plasmids were purified from NEB5-alpha (pCKT-*Chimera*) strains and electroporated into MG1655 cells. After confirming the presence of the plasmid in the new host, MG1655 (pCKT-*Chimera*) was electroporated with the reporter plasmid pHC_DYOLacI-R. Validated MG1655 (pCKT-*Chimera*, pHC_DYOLacI-R) strains were used for *in vivo* and *in vitro* analyses of the functionality of the novel chimeric TFs when required. The sequences of all the oligonucleotides used in this work are found in Table S3.

### Electrophoretic mobility shift assay

To obtain cell extracts enriched in the Sphnx/Siren proteins, NEB5-alpha (pCKT-Sphnx) and NEB5-alpha (pCKT-Siren) cells were grown on LB supplemented with aTc for 5 h at 37˚C in a shaker incubator. Cells were recovered by centrifugation at 4°C (15′ at 4,000 × *g*) and then resuspended in 20 mM Tris-HCl (pH 7.0) to be sonicated at 70 amp for 30 s (30 cycles of 1 s; Branson Digital Sonifier, Danbury, CN). A subsequent centrifugation at 4°C (20′ at 20,000 × *g*) was performed to eliminate cell debris from the sample. The recovered supernatant was then incubated with the DNA probe, a 251 bp dsDNA fragment amplified from plasmid pHC_DYOLacI-R with primers SV00028/SV00029, containing *P_lac_* (including *lacO*). Cell extract (5 μL) and DNA probe (5 μL, final concentration in reaction 50 nM) were incubated together with reaction buffer (20% glycerol, 100 mM KCl, 500 μg·mL^−1^ bovine serum albumin, 20 mM Tris-HCl, pH 7.5). The reaction mixture is incubated for 30′ at 30˚C. After incubation of the retardation mixtures, 2 µL of Gel loading dye without SDS (NEB, Ipswich, MA) was added to each reaction. Mixtures were subsequently fractionated by electrophoresis in precast 6% polyacrylamide gels (Novex TBE Gels; Invitrogen, Waltham, MA) buffered with 0.5× TBE (45 mM Tris borate, 1 mM EDTA). Gels were run at constant voltage (100 V) for 90′ in a Mini Gel Tank (Invitrogen, Waltham, MA) and afterwards stained for 1 h in 50 mL of 0.5× TBE buffer supplemented with 5 µL of SYBR Gold dye (Invitrogen, Waltham, MA) while gently shaken. Post-staining, gels were imaged in a UVP UVSolo Touch (Analytic Jena, Jena, Germany).

### qRT-PCR analysis

Quantitative real-time PCR (qRT-PCR) was used to assay the transcription of our reporter gene (*sfgfp*). MG1655 (pCKT-*Chimera*, pHCDYOLacI-R) strains were precultured overnight in 3 mL LB supplemented with the corresponding selection antibiotics. On the next day, 100 µL of overnight preculture was used as inoculum for several cultures, 10 mL each, of fresh LB (plus selection antibiotics). Each one of the cultures was supplemented so that it would represent one of these three different conditions: Basal (aTc^-^, Neu5Ac^-^), Repressed (aTc^+^, Neu5Ac^-^), and De-repressed (aTc^+^, Neu5Ac^+^). Where experimental conditions required it, cultures were supplemented with 0.5 μg·mL^−1^ anhydrotetracycline (aTc) (Clontech, Mountain View, CA) and 1 mM neuraminic acid (Neu5Ac; Sigma, St. Louis, MO). For every independent qRT-PCR experiment, three biological replicates were created across each of these three conditions. These cultures were placed in a shaker incubator at 37˚C for 5 h and pelleted at 4˚C and 4,000 × *g*. Pellets were either frozen at −80˚C for future use or immediately processed for total RNA extraction. Cells from each individual pellet were set for lysis by resuspension in 1 mL of IBI reagent, a phenol and guanidine isothiocyanate mixture (IBI Scientific, Dubuque, IA). 200 µL of chloroform (Fisher Scientific, Pittsburgh, PA) was added to the lysate, and the mixture was vigorously shaken to homogeneity, followed by centrifugation (benchtop minifuge) at 4˚C and 14,500 rpm for 15 min that led to a phase separation. 500 µL of the upper aqueous phase was mixed with an equal volume of 100% ethanol. At this point, total RNA was purified from the DNA/RNA phase with the Direct-zol RNA Miniprep Kit (Zymo Research, Irvine, CA), where the TRIzol reagent steps are replaced with the phenol and chloroform phase separation. Total RNA eluted with ddH_2_O was then retrotranscribed using qScript Flex cDNA Synthesis Kit (QuantaBio, Beverly, MA). A volume totaling 1 µg RNA was incubated with random hexamers and then supplemented with retrotranscriptase enzyme mix to synthesize cDNA. cDNA from the last step was used as a template for the amplification of a 256 bp fragment with oligonucleotides SV00027A/SV00027 in a CFX96 Real-Time PCR System (Bio-Rad, Hercules, CA). Primers had been designed to amplify a region of pHC_DYOLacI comprising the beginning of *sfgfp*, so that when the tested chimera was being expressed, it repressed the expression of GFP unless Neu5Ac was present. Cq values were analyzed as a measure of gene expression using the ΔΔCq (2^−ΔΔCt^) method (137).

### Protein structure prediction using AlphaFold 3

The 3D structures of the target proteins, Sphnx (LacI-LNK2-SiaP, N_t_-DBD-EKEKEK-LBD-C_t_) and Kunst (LacI-LNK3-SiaP, N_t_-DBD-GSGSGS-LBD-C_t_), were predicted using AlphaFold 3 (121), developed by Google DeepMind (London, UK). The predictions were performed using the publicly available AlphaFold 3 web server (https://golgi.sandbox.google.com/). Both Sphnx and Kunst proteins were modeled as dimers, along with the DNA double helix containing *lacO* (5′-ATTGTGAGCGGATAACAATT-3′ and its complementary sequence). Post-prediction, the structures were visualized and further analyzed using VMD (138) to determine key protein-protein interactions.

### Molecular docking simulations

Sphnx (LacI-LNK2-SiaP) and Kunst 3D structures were used to forecast the primary binding patterns between the TF and a collection of putative ligands: Neu5Ac (PubChem CID 439197), ManNAc (PubChem CID 439281), GlcNAc (PubChem CID 439174), and Neu5Gc (PubChem CID 440001). First, water molecules and heteroatoms were removed from the PDB files for Sphnx and Kunst 3D structures. Using AutoDock4 (139), Kollman charges were supplemented to the 3D structure of both our ligands (see PubChem reference above) and proteins, with polar hydrogen charges also being added to the transcription factor structures. Once ligands and receptors were prepared, a grid box delineating the region of the whole protein where the docking would take place was established. Employing the molecular docking simulation protocols provided by the AutoDock 4 suite, binding affinities were assessed. Post-docking, visualization software (Pymol, Schrödinger Inc., New York, NY; Discovery Studio, Biovia, San Diego, CA) was used to identify interactions between Sphnx/Kunst and the aforementioned ligands.

### Statistical analysis and data visualization

Statistical analyses and data visualization were performed in R (https://www.r-project.org/) using the tidyverse (140), ggplot2 (141), and data.table (142) ecosystems within the RStudio (RStudio Inc., Boston, MA) and Visual Studio Code environments (Microsoft Corp., Redmond, WA). Raw plate-reader measurements (OD_600_ and GFP-associated fluorescence) were imported directly from instrument-generated Microsoft Excel files (Microsoft Corp., Redmond, WA). For each experimental run, the background signal was removed by subtracting the mean fluorescence of designated blank wells at each timepoint. For conditions measured in technical replicates, replicate values were aggregated by computing the mean and standard deviation at each timepoint. To account for differences in culture growth, relative fluorescence (Rel.fluor) was calculated as GFP-associated fluorescence in arbitrary units normalized to OD_600_ and scaled by a constant factor (fluorescence/OD_600_ × 1,000). Time-course data were visualized using the ggplot2 package. For each condition, time-resolved trajectories were plotted as mean values with error bars representing ±1 standard deviation across technical replicates. For bioreplicate analyses, processed data files from independent experimental runs were imported and harmonized by matching condition names and aligning shared timepoints across runs. For each condition and timepoint, the mean and standard deviation across biological replicates were computed. These aggregated values were visualized as mean trajectories with standard-deviation error bars, using identical normalization and scaling procedures as described above. Subset analyses were performed by selecting user-defined groups of conditions and generating separate visualizations for each group. Comparisons in Rel.fluor between strains grown under different conditions were performed with Welch’s one-way ANOVA followed by pair-wise *t*-tests (Fig. 2b and 3b). In the case of Fig. S1, hourly OD_600_ values for supplemented strains were compared to those of the unsupplemented control via pair-wise t-tests. For Fig. S4, R_Induced/Repressed_, expressing the difference between induced and repressed conditions, was tested against the null hypothesis using a chi-squared test.

## SUPPLEMENTAL MATERIAL

Supplemental material aem.02027-25-s0001.docx Tables S1 to S4, Fig. S1 to S6, and transcription factor sequences.

ASM does not own the copyrights to Supplemental Material that may be linked to, or accessed through, an article. The authors have granted ASM a non-exclusive, world-wide license to publish the Supplemental Material files. Please contact the corresponding author directly for reuse.
