# Supplementary material for: Expanding salivary biomarker detection by creating a synthetic neuraminic acid sensor via chimeragenesis
Source: Appl Environ Microbiol. 2026 Apr 29;92(5):e02027-25. doi: 10.1128/aem.02027-25 (PMC13188862; doi:10.1128/aem.02027-25)
Supplement: Supplemental material — Tables S1 to S4, Fig. S1 to S6, and transcription factor sequences. [file aem.02027-25-s0001.docx]

**SUPPLEMENTARY MATERIALS**

Expanding salivary biomarker detection by creating a synthetic neuraminic acid sensor via chimeragenesis

Samuel J. Verzino^1^, Sharona A. Priyev^1#^, Valeria A. Sánchez Estrada^1,3#^, Ali GholamianMogaddan^1#^, Gemma X. Crowley^1^, Alexandra Rutkowski^1^, Amelia C. Lam^1^, Elizabeth S. Nazginov^1^, Paola Kotemelo^1^, Agustina Bacelo^1,2^, Jack D. Flannery^1^, Ksenya Gavrilov^1^, Desiree T. Sukhram^1^, Frank X. Vázquez^2^, Javier F. Juárez^1^

^1^Dept. of Biological Sciences and ^2^Dept. of Chemistry, Saint John’s University, 8000 Utopia Parkway, Queens, NY (USA)

^3^Biological and Medical Informatics, Graduate Division, University of California San Francisco (UCSF), 500 Parnassus Avenue, San Francisco, CA 94143

**INDEX**

**Supplementary Tables**

- ***Supplementary Table 1.*** Linker (LNK) regions used in this study.
- ***Supplementary Table 2.*** Bacterials strains and plasmids used in this study.
- ***Supplementary Table 3.*** Oligonucleotides used in this study.
- ***Supplementary Table 4.*** Relative fluorescence over time of different MG1655 (pCKT-*Chimera*, pHC_DYOLacI-R) strains.

**Supplementary Figures**

- ***Supplementary Figure 1*.** Growth of MG1655 (pHC_DYOLacI-R) supplemented with different concentrations of neuraminic acid.
- ***Supplementary Figure 2*.** *In vivo* behaviour of MG1655 (pCKT-Siren, pHC_DYOLacI-R) strain expressing Siren (LacI-LNK1-SiaP).
- ***Supplementary Figure 3*.** *In vivo* behaviour of MG1655 (pCKT-Kunst, pHC_DYOLacI-,) strain expressing Kunst (LacI-LNK3-SiaP).
- ***Supplementary Figure 4***. qRT-PCR analysis of the regulated expression of GFP by Sphnx.
- ***Supplementary Figure 5***. Electrophoresis mobility assay Sphnx-*P_lac_*.
- ***Supplementary Figure 6.*** Relative fluorescence of MG-Sphnx cells after induction with Neu5Ac under conditions minimizing cell growth.

**Appendix:** Relevant transcription factor sequences.

# Supplementary Table 1


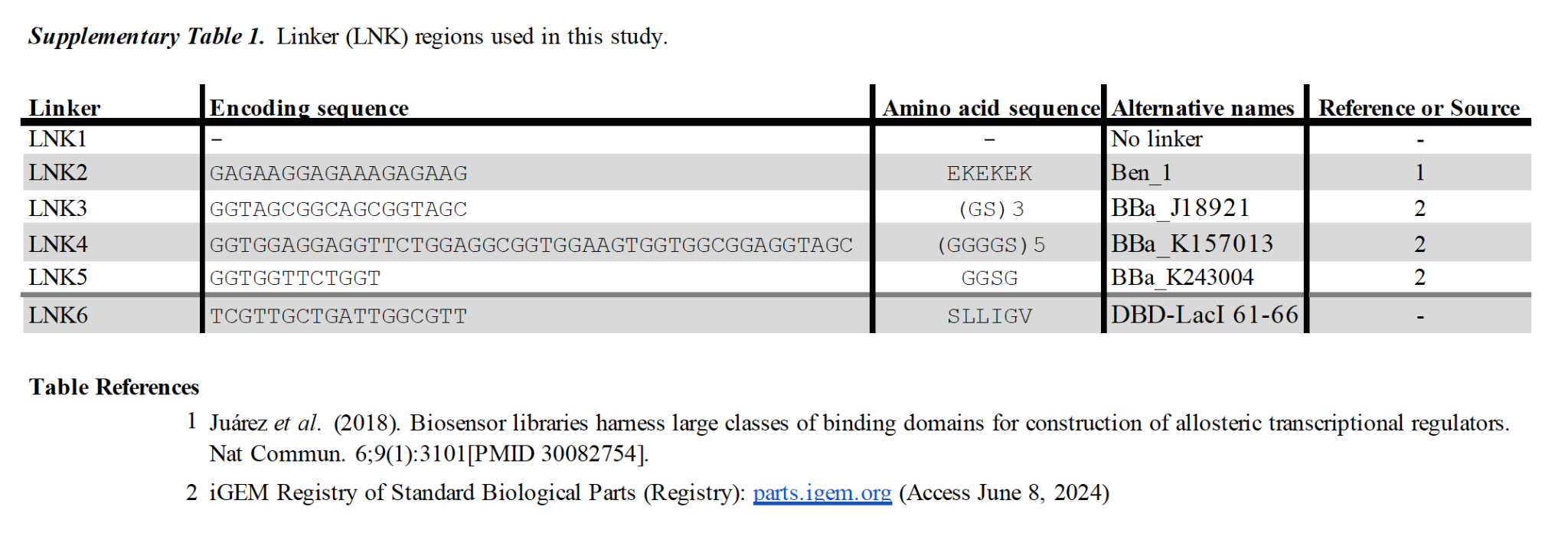


# Supplementary Table 2


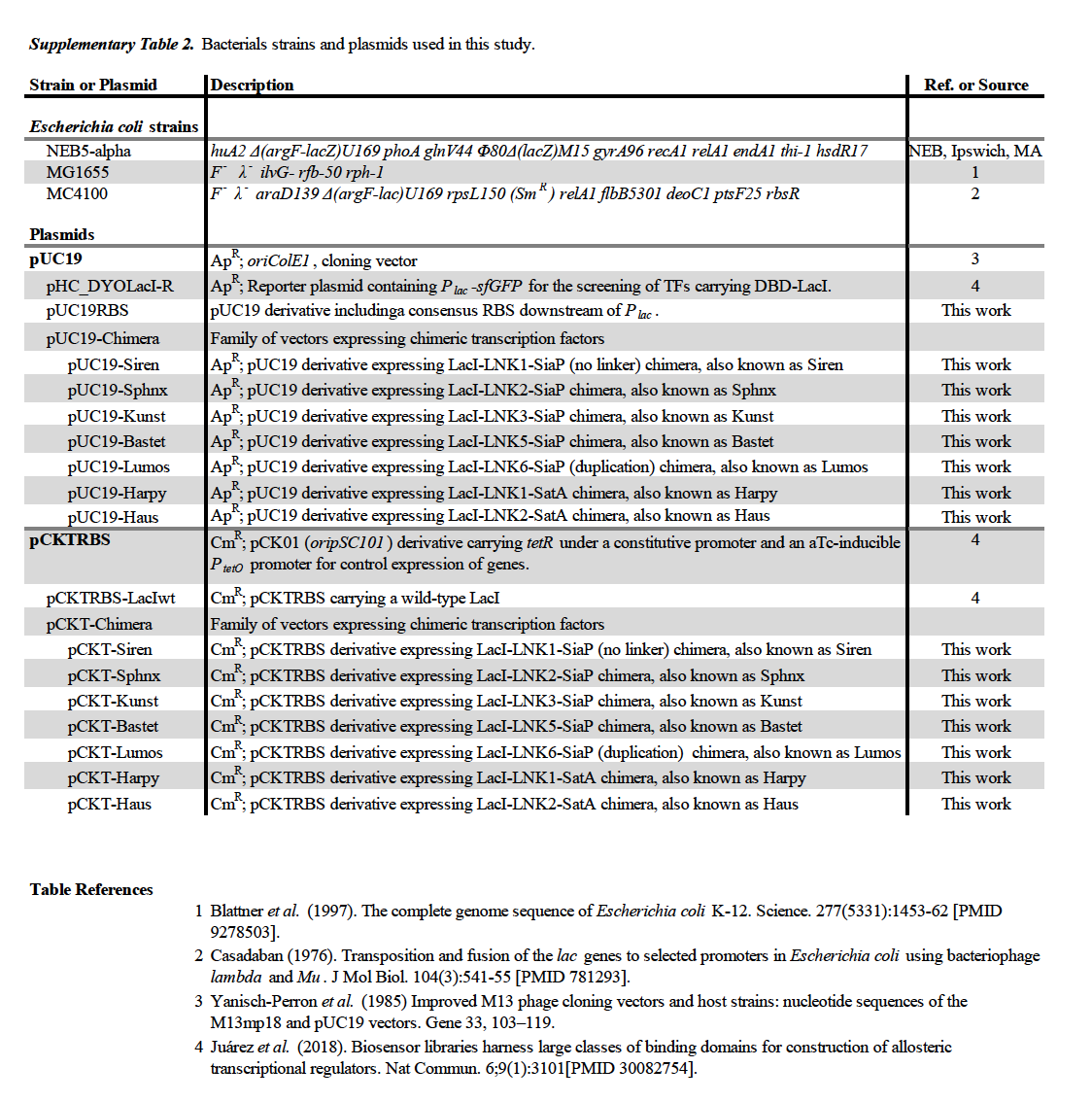


# Supplementary Table 3
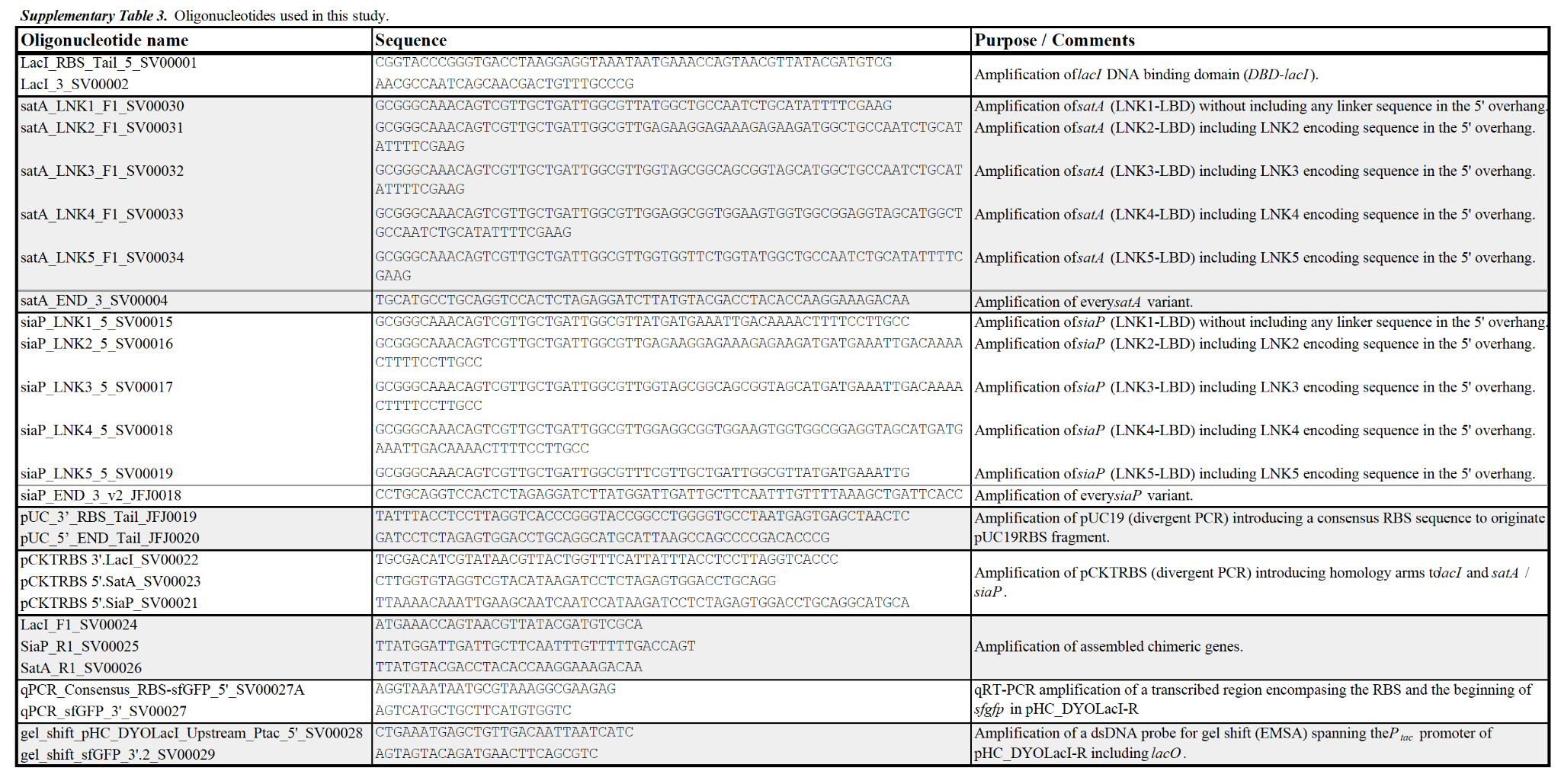


# Supplementary Table 4

| **Name** | |  |
| --- | --- | --- |
| **Common** | **Systematic** | **Graphs** |
| **Siren** | LacI-LNK1-SiaP | 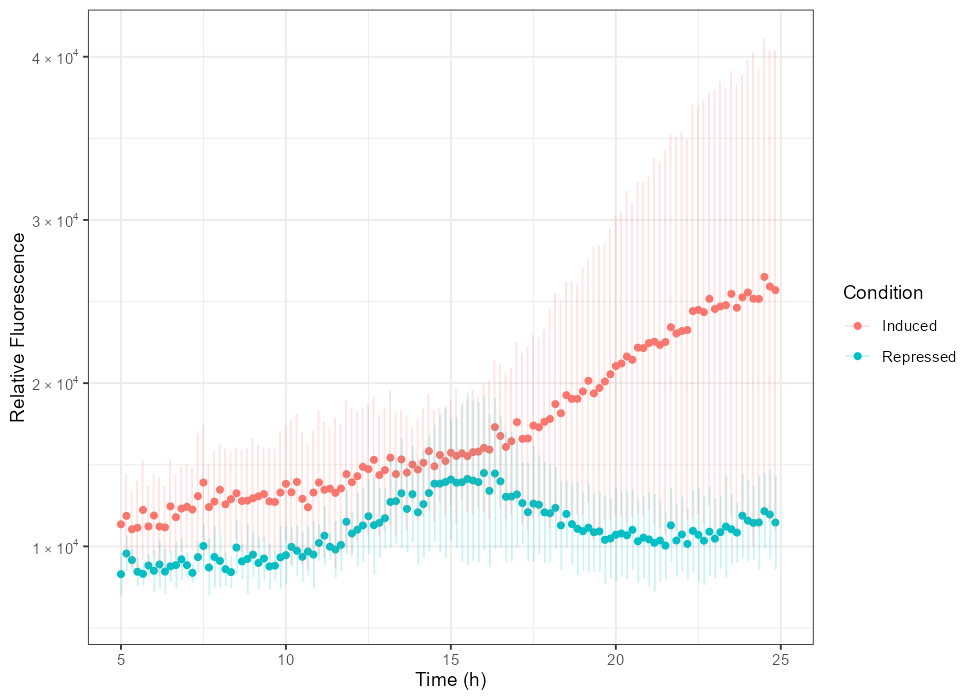 |
| **Sphnx** | LacI-LNK2-SiaP | 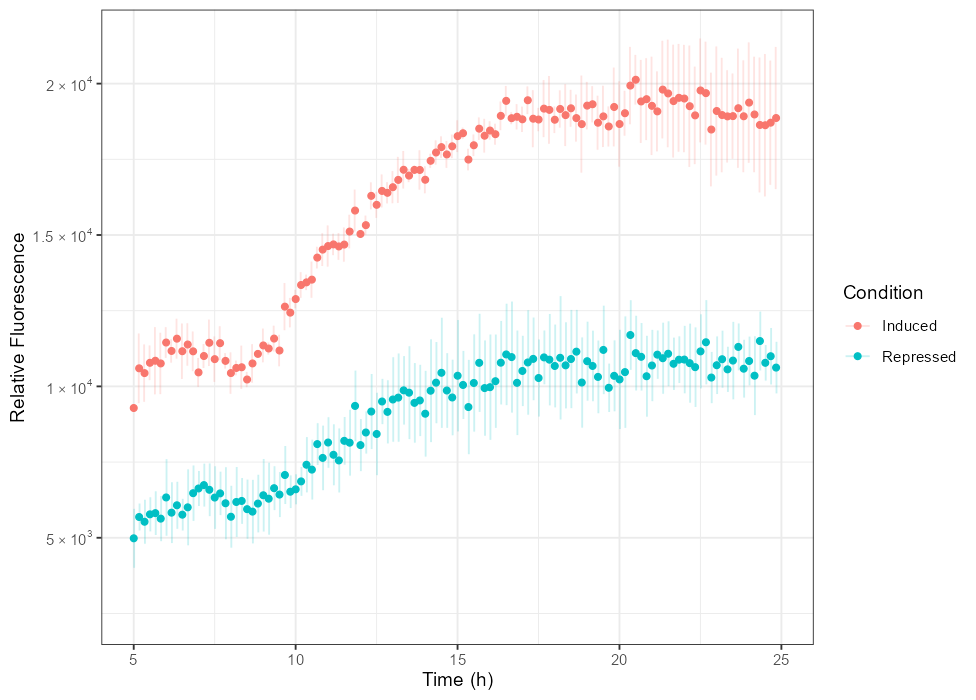 |
| **Kunst** | LacI-LNK3-SiaP | 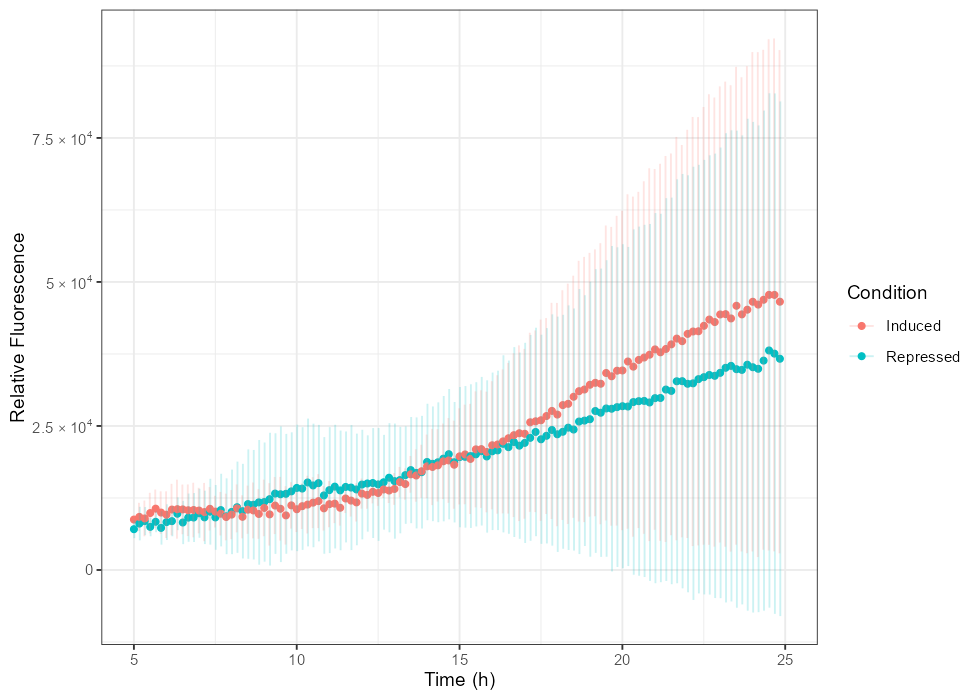 |
| **Scorpio** | LacI-LNK4-SiaP | 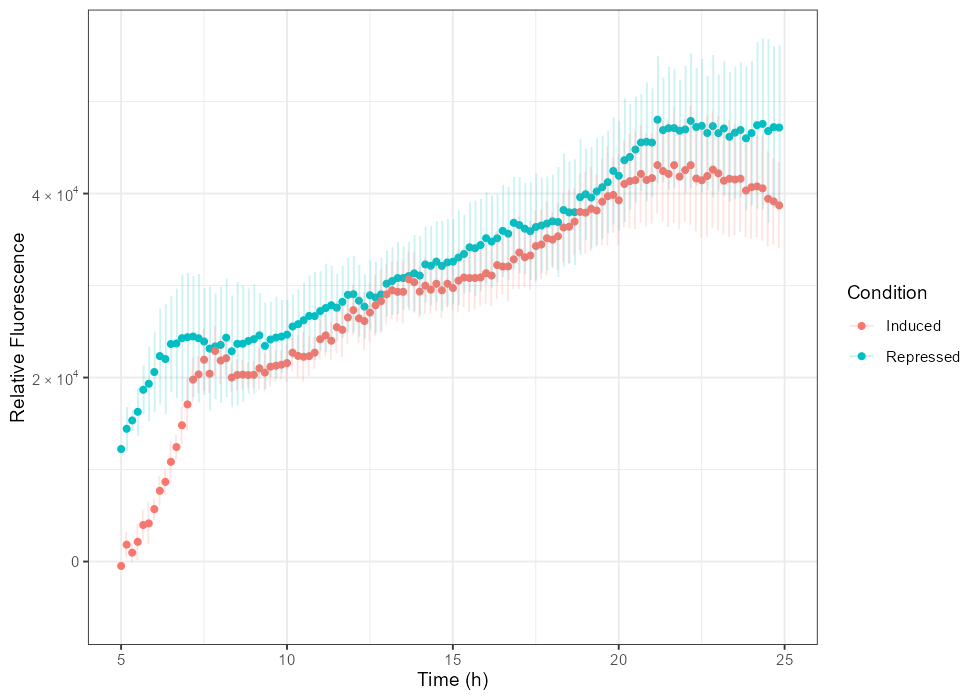 |
| **Bastet** | LacI-LNK5-SiaP | 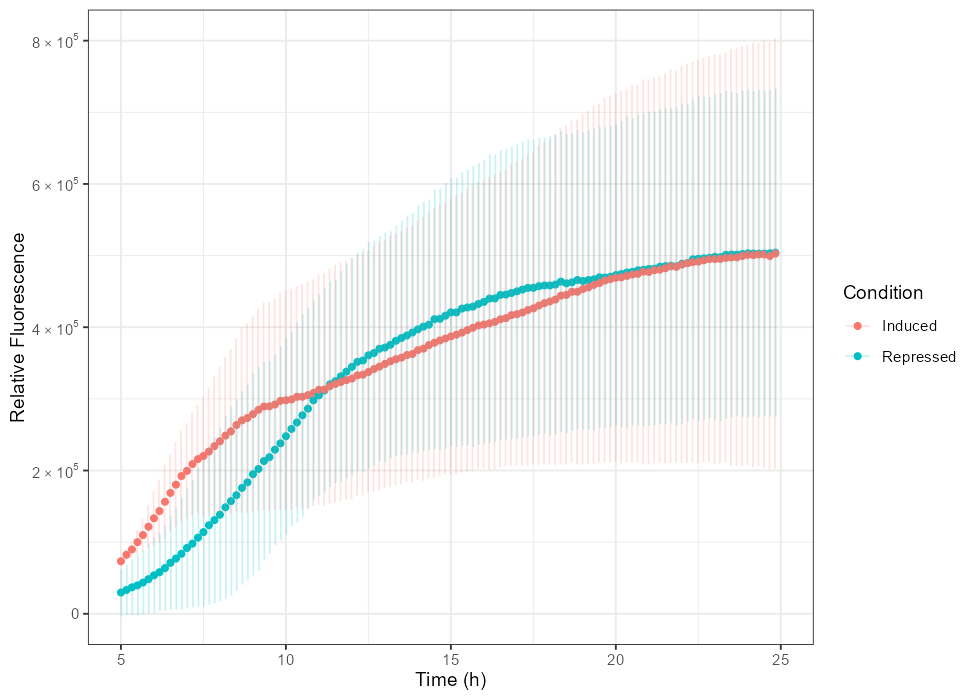 |
| **Lumos** | LacI-LNK6-SiaP | 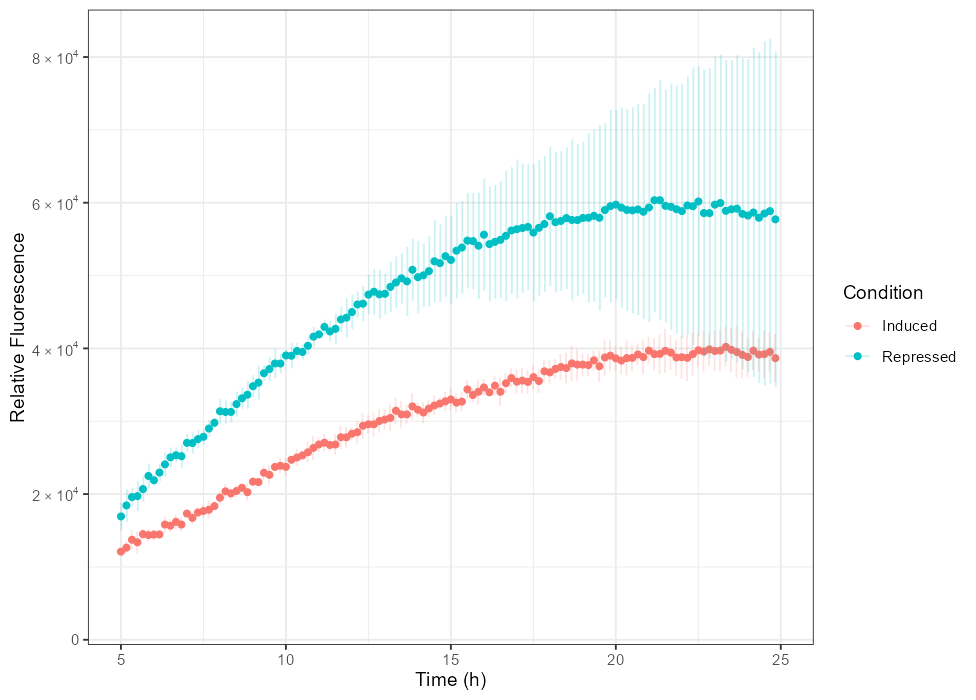 |
| **Harpy** | LacI-LNK1-SatA | 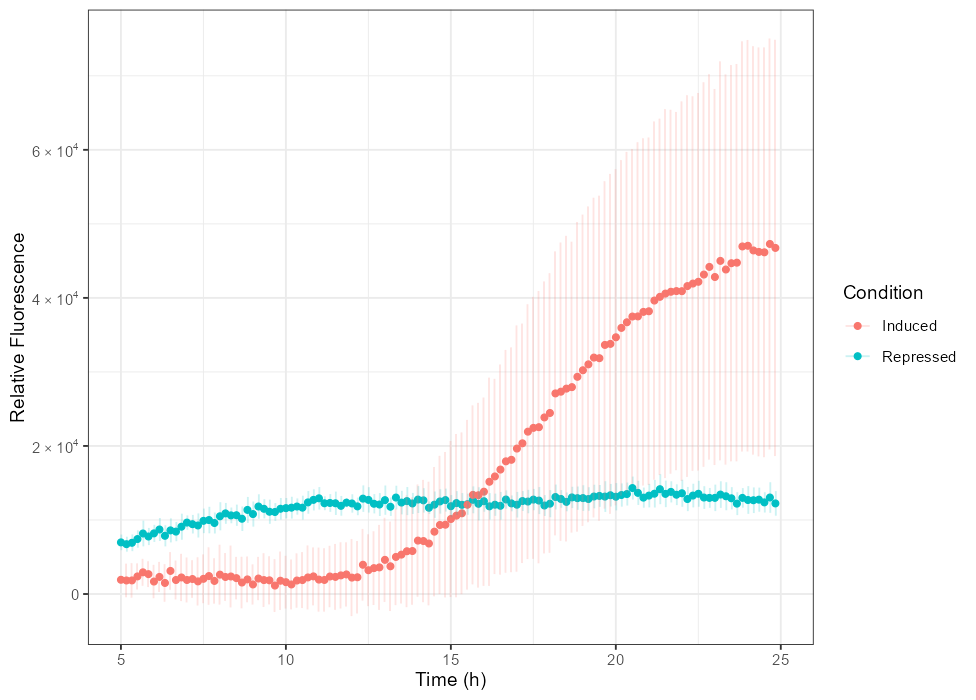 |
| **Haus** | LacI-LNK2-SatA | 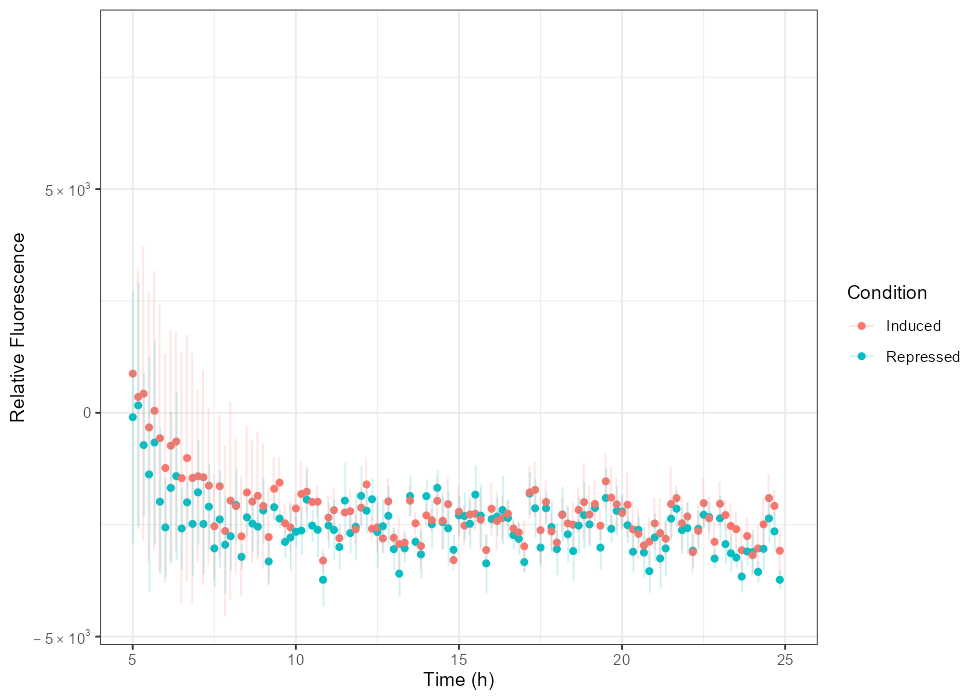 |

***Supplementary Table 4.*** Relative fluorescence over time of different MG1655 (pCKT-*Chimera*, pHC_DYOLacI-R) strains. The first two columns of this summary table includes both the systematic and common names assigned to every chimeric TF created in this work. The third column displays a representative time course (hours) showing relative fluorescence of MG1655 (pCKT-*Chimera*, pHC_DYOLacI-R) cells grown in a multiwell plate reader, with error bars denoting SEM (standard error of the mean) associated to technical replicas (*n* = 4). Promoter activity is measured as relative fluorescence (GFP-associated fluorescence in arbitrary units / OD_600_) of the strain growing in the de-repressed (aTc^+^, Neu5Ac^+^; *orange dots*) or repressed (aTc^+^, Neu5Ac^-^; *blue dots*) states.

#

# Supplementary Figure 1
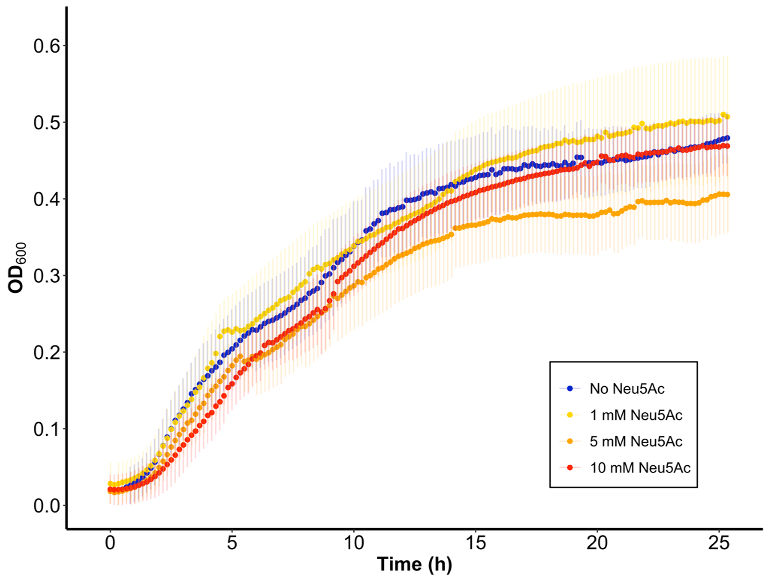


***Supplementary Figure 1*.** Growth of MG1655 (pHC_DYOLacI-R) in LB media (*green dots*) or in LB media supplemented with neuraminic acid at concentrations: 1 mM (*yellow dots*), 5 mM (*orange dots*), 10 mM (*red dots*). Bacterial cultures were grown and their OD_600_ and GFP-associated fluorescence tracked over time in a multiwell plate reader as described in *Materials and Methods.* Error bars denote SEM (standard error of the mean) associated to independent biological replicas (*n* = 3).

# Supplementary Figure 2


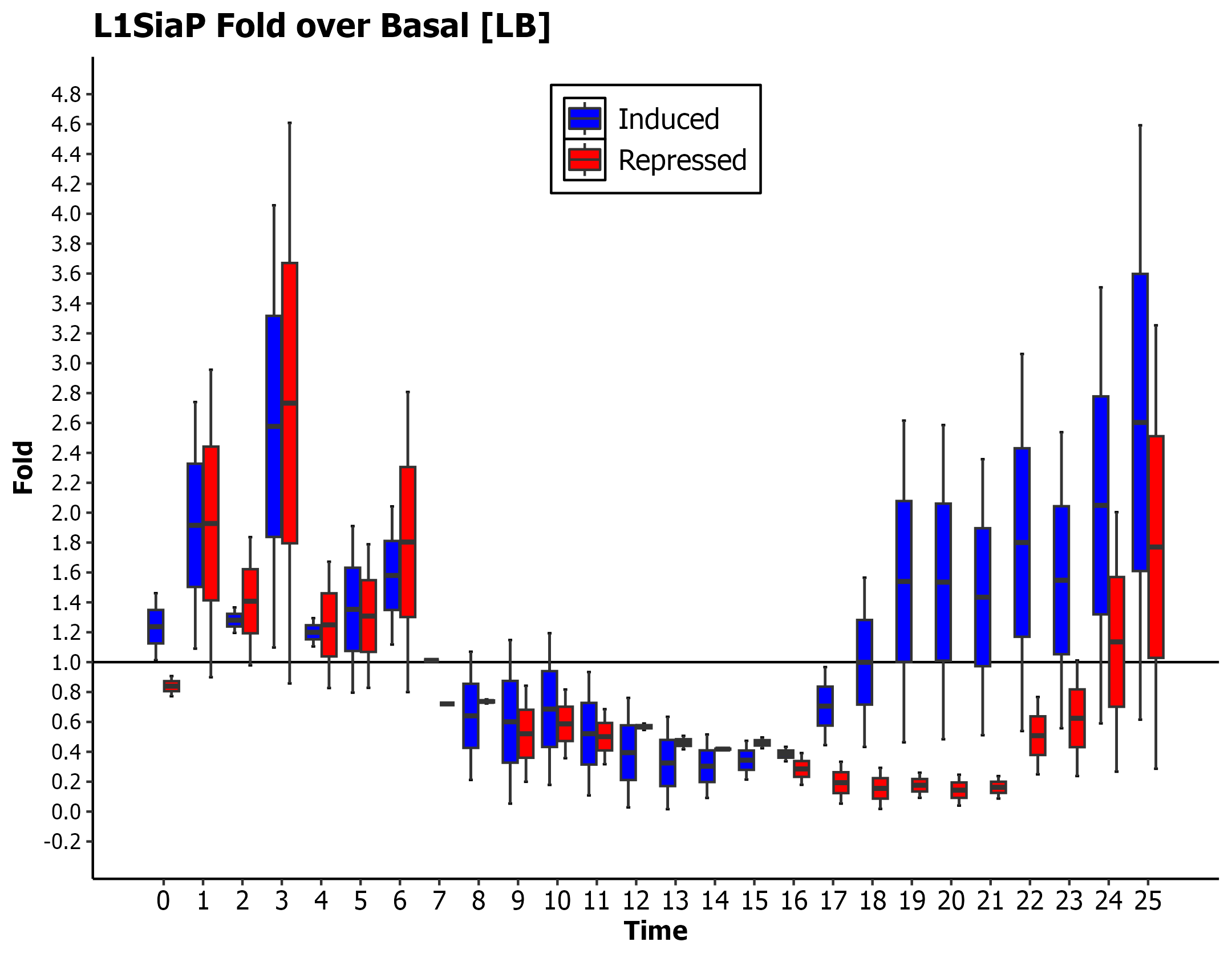


***Supplementary Figure 2*. *In vivo* behaviour of a strain expressing Siren (LacI-LNK1-SiaP).** Time course (hours) showing relative fluorescence of MG1655 (pCKT-Siren, pHC_DYOLacI-R) cells grown in a multiwell plate reader. Promoter activity is measured as fold of the relative fluorescence (GFP-associated fluorescence in arbitrary units / OD_600_) of the strain growing in the de-repressed (aTc^+^, Neu5Ac^+^; *blue boxplots*) or repressed (aTc^+^, Neu5Ac^-^; *red boxplots*) states compared to the basal expression of the reporter (aTc^-^, Neu5Ac^-^). Boxplots with whiskers represent data dispersion of the average values of biological replicas (*n* = 8). It can be appreciated how in order to approach the basal activity (fold = 1) around 17 - 21 h, the addition of neuraminic acid is necessary when the chimera gets expressed by the supplementation of aTc. Growth conditions and fluorescence assays performed as described in *Materials and Methods*.

# Supplementary Figure 3.


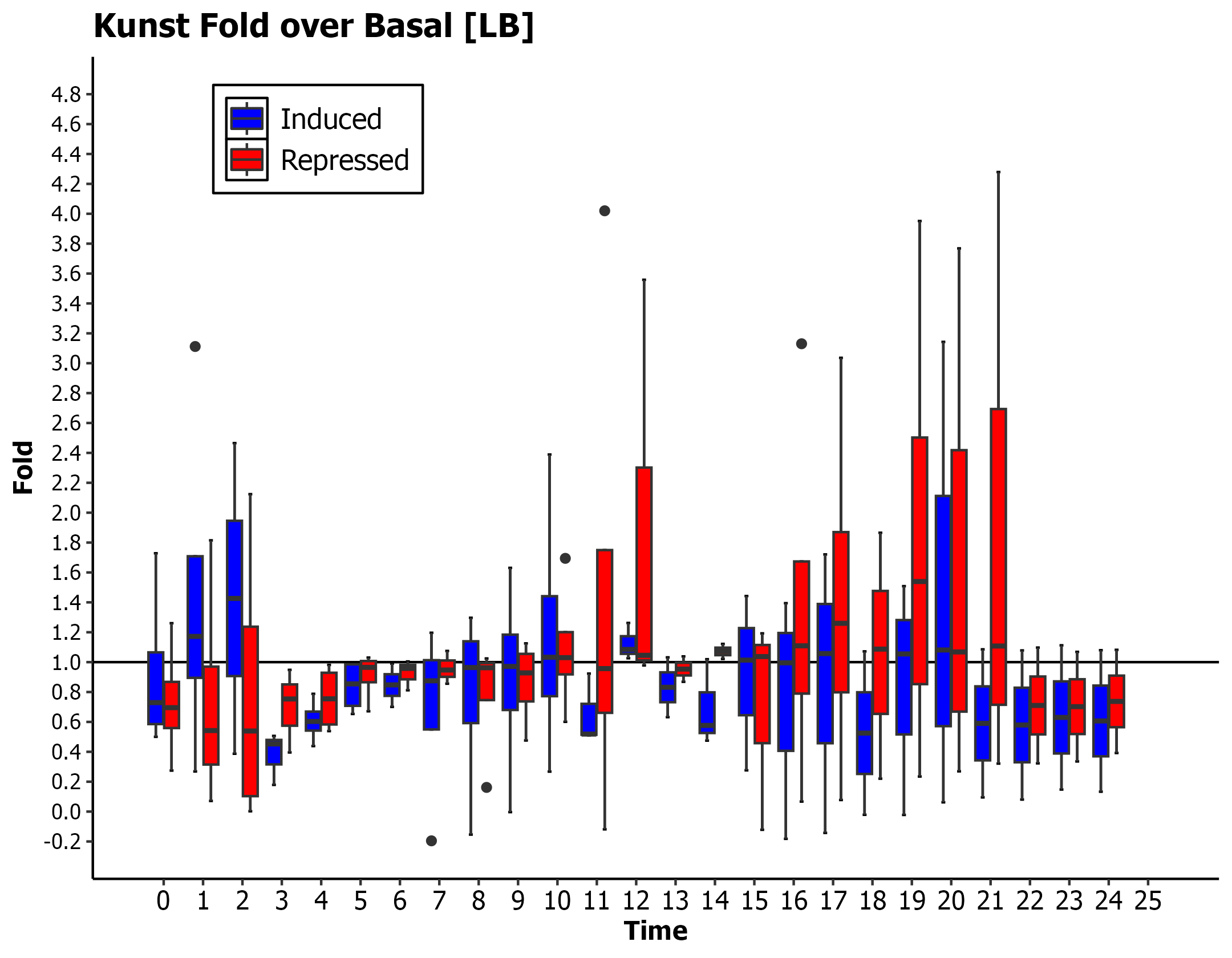


***Supplementary Figure 3***. ***In vivo* behaviour of a strain expressing Kunst (LacI-LNK3-SiaP).** Time course (hours) showing relative fluorescence of MG1655 (pCKT-Kunst, pHC_DYOLacI-R) cells grown in a multiwell plate reader. Promoter activity is measured as fold of the relative fluorescence (GFP-associated fluorescence in arbitrary units / OD_600_) of the strain growing in the de-repressed (aTc^+^, Neu5Ac^+^; *blue boxplots*) or repressed (aTc^+^, Neu5Ac^-^; *red boxplots*) states compared to the basal expression of the reporter (aTc^-^, Neu5Ac^-^). Boxplots with whiskers represent data dispersion of the average values of biological replicas (*n* = 8). It can be appreciated how the strain is not able to display GFP-associated fluorescence in the presence of neuraminic acid. Growth conditions and fluorescence assays performed as described in *Materials and* *Methods*.

# Supplementary Figure 4

#
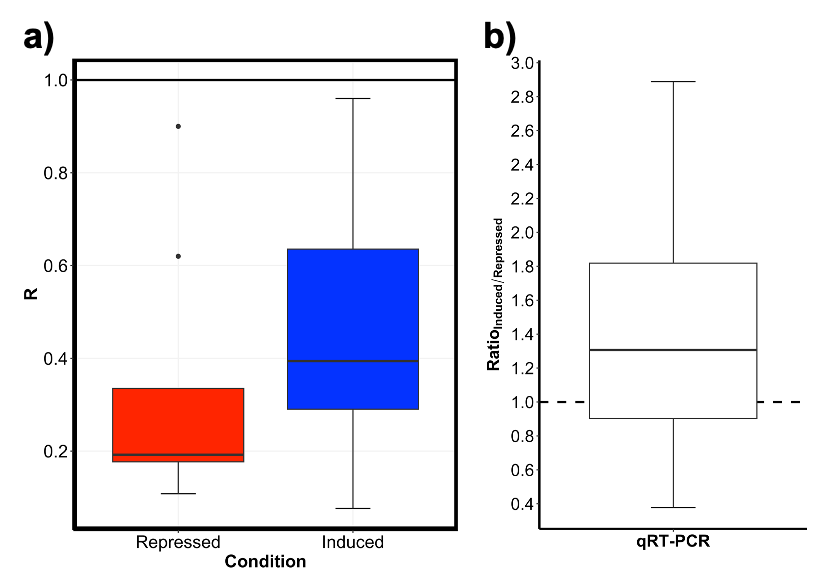


# ***﻿Supplementary Figure 4*.** Relative activity of the *P_lac_* promoter, regulated by Sphnx, measured by quantitative real-time PCR (qRT-PCR). Total RNA was isolated from MG1655 (pCKT-Sphnx, pHCDYOLacI-R) cultures performed as detailed in *Materials and Methods*. **a)** Boxplot depicting the relative activity of the *P_lac_* promoter expressed as fold gene expression (R = 2^-ΔΔCt^) in cells grown under repression (aTc^+^/Neu5Ac^-^; *red*) or de-repression (aTc^+^/Neu5Ac^+^; *blue*) conditions using the basal state (aTc^-^/Neu5Ac^-^) as a reference (*n* = 8). **b)** Graph showing the induced to repressed ratio (R_induced_ / R_repressed_) for the data displayed in panel a).

# Supplementary Figure 5


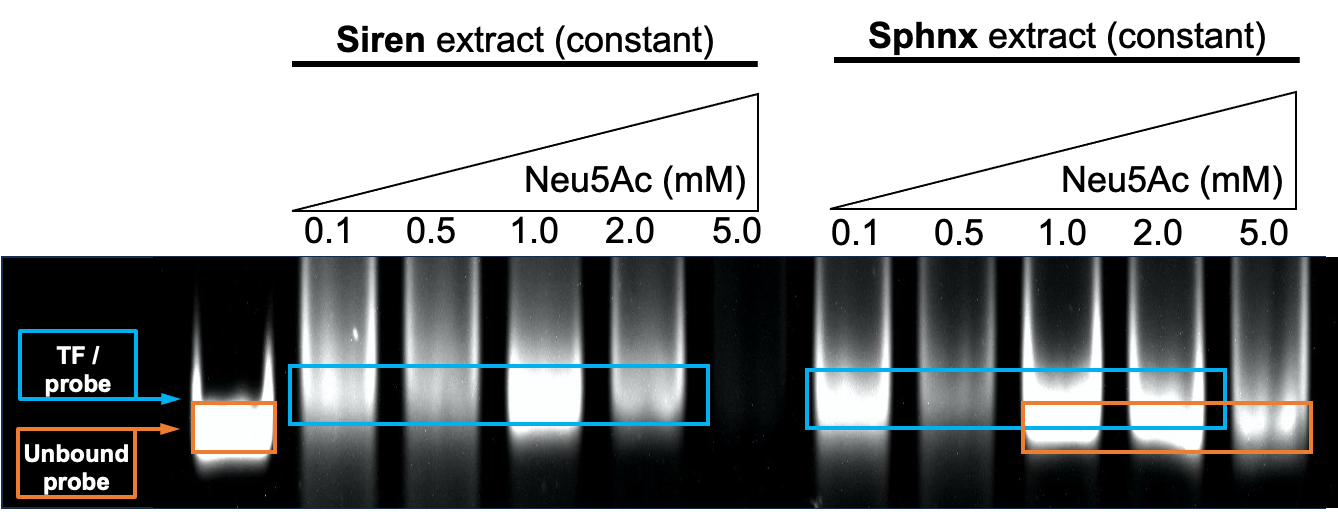


***Supplementary Figure 5.*** **Preliminary *in vitro* testing of the functionality of Sphnx (LacI-LNK2-SiaP).** Assessment of the ability of Sphnx (LacI-LNK2-SiaP) and Siren (LacI-LNK2-SiaP) proteins to interact with their cognate promoter *P_lac_*. ﻿EMSA assays were performed as indicated under *Materials and Methods*. Interaction between NEB5-alpha (pCKT-Chimera) cell extracts expressing either Sphnx or Siren chimeric TFs and a dsDNA probe (*P_lac_-sfgfp*, 251 bp) including the *P_lac_* promoter and the beginning of the sfGFP reporter. A fixed concentration of cell extract and DNA probe was exposed to growing concentrations of Neu5Ac (mM). The positions in the gel associated with unbound and bound probes are identified with orange and blue rectangles, respectively.

Supplementary Figure 6


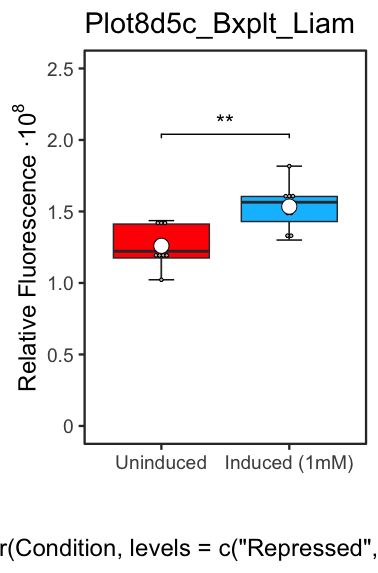


***﻿Supplementary Figure 6*.** **Relative fluorescence of MG-Sphnx cells after induction with Neu5Ac under conditions minimizing cell growth after 4 h of incubation.** Promoter activity is measured as Rel.fluor, relative fluorescence (GFP-associated fluorescence in arbitrary units / OD600 × 1000) of the strain growing in repressed (aTc^+^, Neu5Ac^-^; *red boxplot*) or de-repressed (aTc^+^, Neu5Ac^+^; *blue boxplot*) conditions. 1 mM Neu5Ac was added to the de-repressed cell preparations. Boxplots with whiskers represent data dispersion of the average values of biological replicas (n = 8; average of the means, *white circle*). Experimental conditions described in *Materials and Methods*. Statistically significant differences between selected conditions are marked with stars (** *p-value* < 0.01).

# Appendix: Relevant transcription factor sequences

Common name: **Sphnx**.

Systematic name: **LacI-LNK2-SiaP** (N_t_-DBD-LNK-LBD-C_t_)

>*sphnx*_nt_sequence (1206 bp)

ATGAAACCAGTAACGTTATACGATGTCGCAGAGTATGCCGGTGTCTCTTATCAGACCGTTTCCCGCGTGGTGAACCAGGCCAGCCACGTTTCTGCGAAAACGCGGGAAAAAGTGGAAGCGGCGATGGCGGAGCTGAATTACATTCCCAACCGCGTGGCACAACAACTGGCGGGCAAACAGTCGTTGCTGATTGGCGTTGAGAAGGAGAAAGAGAAGATGATGAAATTGACAAAACTTTTCCTTGCCACCGCCATTTCTTTAGGCGTATCTTCTGCTGTTCTTGCCGCTGATTATGACTTGAAATTCGGTATGAATGCTGGAACTTCATCAAATGAATATAAAGCGGCAGAAATGTTTGCCAAAGAAGTCAAAGAAAAATCACAGGGTAAAATTGAAATTTCACTTTATCCAAGTTCACAATTAGGTGATGACCGTGCAATGTTAAAACAATTAAAAGACGGTTCTCTCGACTTTACCTTTGCAGAATCTGCTCGCTTCCAGCTGTTTTACCCTGAAGCGGCAGTATTTGCCTTACCTTATGTTATTAGCAACTACAATGTTGCACAAAAAGCCTTATTCGATACAGAATTCGGTAAAGATTTAATTAAAAAAATGGATAAAGATCTTGGCGTGACTTTACTTTCCCAAGCTTATAACGGAACTCGCCAAACGACTTCAAATCGTGCAATCAACAGTATTGCAGATATGAAAGGCTTAAAACTTCGTGTGCCAAATGCAGCAACAAACTTAGCCTATGCTAAATATGTTGGTGCATCACCAACACCAATGGCATTTTCTGAAGTTTATCTTGCGTTACAAACCAATGCCGTCGATGGTCAAGAAAACCCGTTAGCAGCGGTGCAAGCACAAAAATTCTATGAAGTGCAAAAGTTCTTAGCAATGACTAATCATATTTTGAATGACCAACTTTATTTAGTAAGCAACGAGACTTATAAAGAACTCCCTGAAGATCTTCAAAAAGTCGTAAAAGATGCTGCCGAAAATGCAGCAAAATATCACACTAAATTATTCGTAGATGGAGAGAAAGATTTAGTCACATTCTTTGAAAAACAAGGCGTGAAAATTACACATCCTGATCTTGTTCCATTTAAAGAATCAATGAAGCCGTATTATGCTGAGTTTGTAAAACAAACTGGTCAAAAAGGTGAATCAGCTTTAAAACAAATTGAAGCAATCAATCCATAA

>Sphnx_aa_sequence (401 aa)

MKPVTLYDVAEYAGVSYQTVSRVVNQASHVSAKTREKVEAAMAELNYIPNRVAQQLAGKQSLLIGVEKEKEKMMKLTKLFLATAISLGVSSAVLAADYDLKFGMNAGTSSNEYKAAEMFAKEVKEKSQGKIEISLYPSSQLGDDRAMLKQLKDGSLDFTFAESARFQLFYPEAAVFALPYVISNYNVAQKALFDTEFGKDLIKKMDKDLGVTLLSQAYNGTRQTTSNRAINSIADMKGLKLRVPNAATNLAYAKYVGASPTPMAFSEVYLALQTNAVDGQENPLAAVQAQKFYEVQKFLAMTNHILNDQLYLVSNETYKELPEDLQKVVKDAAENAAKYHTKLFVDGEKDLVTFFEKQGVKITHPDLVPFKESMKPYYAEFVKQTGQKGESALKQIEAINP

Common name: **Kunst**.

Systematic name: **LacI-LNK3-SiaP** (N_t_-DBD-LNK-LBD-C_t_)

>*kunst*_nt_sequence (1206 bp)

ATGAAACCAGTAACGTTATACGATGTCGCAGAGTATGCCGGTGTCTCTTATCAGACCGTTTCCCGCGTGGTGAACCAGGCCAGCCACGTTTCTGCGAAAACGCGGGAAAAAGTGGAAGCGGCGATGGCGGAGCTGAATTACATTCCCAACCGCGTGGCACAACAACTGGCGGGCAAACAGTCGTTGCTGATTGGCGTTGGTAGCGGCAGCGGTAGCATGATGAAATTGACAAAACTTTTCCTTGCCACCGCCATTTCTTTAGGCGTATCTTCTGCTGTTCTTGCCGCTGATTATGACTTGAAATTCGGTATGAATGCTGGAACTTCATCAAATGAATATAAAGCGGCAGAAATGTTTGCCAAAGAAGTCAAAGAAAAATCACAGGGTAAAATTGAAATTTCACTTTATCCAAGTTCACAATTAGGTGATGACCGTGCAATGTTAAAACAATTAAAAGACGGTTCTCTCGACTTTACCTTTGCAGAATCTGCTCGCTTCCAGCTGTTTTACCCTGAAGCGGCAGTATTTGCCTTACCTTATGTTATTAGCAACTACAATGTTGCACAAAAAGCCTTATTCGATACAGAATTCGGTAAAGATTTAATTAAAAAAATGGATAAAGATCTTGGCGTGACTTTACTTTCCCAAGCTTATAACGGAACTCGCCAAACGACTTCAAATCGTGCAATCAACAGTATTGCAGATATGAAAGGCTTAAAACTTCGTGTGCCAAATGCAGCAACAAACTTAGCCTATGCTAAATATGTTGGTGCATCACCAACACCAATGGCATTTTCTGAAGTTTATCTTGCGTTACAAACCAATGCCGTCGATGGTCAAGAAAACCCGTTAGCAGCGGTGCAAGCACAAAAATTCTATGAAGTGCAAAAGTTCTTAGCAATGACTAATCATATTTTGAATGACCAACTTTATTTAGTAAGCAACGAGACTTATAAAGAACTCCCTGAAGATCTTCAAAAAGTCGTAAAAGATGCTGCCGAAAATGCAGCAAAATATCACACTAAATTATTCGTAGATGGAGAGAAAGATTTAGTCACATTCTTTGAAAAACAAGGCGTGAAAATTACACATCCTGATCTTGTTCCATTTAAAGAATCAATGAAGCCGTATTATGCTGAGTTTGTAAAACAAACTGGTCAAAAAGGTGAATCAGCTTTAAAACAAATTGAAGCAATCAATCCATAA

>Kunst_aa_sequence (401 aa)

MKPVTLYDVAEYAGVSYQTVSRVVNQASHVSAKTREKVEAAMAELNYIPNRVAQQLAGKQSLLIGVGSGSGSMMKLTKLFLATAISLGVSSAVLAADYDLKFGMNAGTSSNEYKAAEMFAKEVKEKSQGKIEISLYPSSQLGDDRAMLKQLKDGSLDFTFAESARFQLFYPEAAVFALPYVISNYNVAQKALFDTEFGKDLIKKMDKDLGVTLLSQAYNGTRQTTSNRAINSIADMKGLKLRVPNAATNLAYAKYVGASPTPMAFSEVYLALQTNAVDGQENPLAAVQAQKFYEVQKFLAMTNHILNDQLYLVSNETYKELPEDLQKVVKDAAENAAKYHTKLFVDGEKDLVTFFEKQGVKITHPDLVPFKESMKPYYAEFVKQTGQKGESALKQIEAINP

Common name: **Siren**.

Systematic name: **LacI-LNK1-SiaP** (N_t_-DBD-LBD-C_t_), no linker sequence

>*siren*_nt_sequence (1188 bp)

ATGAAACCAGTAACGTTATACGATGTCGCAGAGTATGCCGGTGTCTCTTATCAGACCGTTTCCCGCGTGGTGAACCAGGCCAGCCACGTTTCTGCGAAAACGCGGGAAAAAGTGGAAGCGGCGATGGCGGAGCTGAATTACATTCCCAACCGCGTGGCACAACAACTGGCGGGCAAACAGTCGTTGCTGATTGGCGTTATGATGAAATTGACAAAACTTTTCCTTGCCACCGCCATTTCTTTAGGCGTATCTTCTGCTGTTCTTGCCGCTGATTATGACTTGAAATTCGGTATGAATGCTGGAACTTCATCAAATGAATATAAAGCGGCAGAAATGTTTGCCAAAGAAGTCAAAGAAAAATCACAGGGTAAAATTGAAATTTCACTTTATCCAAGTTCACAATTAGGTGATGACCGTGCAATGTTAAAACAATTAAAAGACGGTTCTCTCGACTTTACCTTTGCAGAATCTGCTCGCTTCCAGCTGTTTTACCCTGAAGCGGCAGTATTTGCCTTACCTTATGTTATTAGCAACTACAATGTTGCACAAAAAGCCTTATTCGATACAGAATTCGGTAAAGATTTAATTAAAAAAATGGATAAAGATCTTGGCGTGACTTTACTTTCCCAAGCTTATAACGGAACTCGCCAAACGACTTCAAATCGTGCAATCAACAGTATTGCAGATATGAAAGGCTTAAAACTTCGTGTGCCAAATGCAGCAACAAACTTAGCCTATGCTAAATATGTTGGTGCATCACCAACACCAATGGCATTTTCTGAAGTTTATCTTGCGTTACAAACCAATGCCGTCGATGGTCAAGAAAACCCGTTAGCAGCGGTGCAAGCACAAAAATTCTATGAAGTGCAAAAGTTCTTAGCAATGACTAATCATATTTTGAATGACCAACTTTATTTAGTAAGCAACGAGACTTATAAAGAACTCCCTGAAGATCTTCAAAAAGTCGTAAAAGATGCTGCCGAAAATGCAGCAAAATATCACACTAAATTATTCGTAGATGGAGAGAAAGATTTAGTCACATTCTTTGAAAAACAAGGCGTGAAAATTACACATCCTGATCTTGTTCCATTTAAAGAATCAATGAAGCCGTATTATGCTGAGTTTGTAAAACAAACTGGTCAAAAAGGTGAATCAGCTTTAAAACAAATTGAAGCAATCAATCCATAA

>Siren_aa_sequence (396 aa)

MKPVTLYDVAEYAGVSYQTVSRVVNQASHVSAKTREKVEAAMAELNYIPNRVAQQLAGKQSLLIGVMMKLTKLFLATAISLGVSSAVLAADYDLKFGMNAGTSSNEYKAAEMFAKEVKEKSQGKIEISLYPSSQLGDDRAMLKQLKDGSLDFTFAESARFQLFYPEAAVFALPYVISNYNVAQKALFDTEFGKDLIKKMDKDLGVTLLSQAYNGTRQTTSNRAINSIADMKGLKLRVPNAATNLAYAKYVGASPTPMAFSEVYLALQTNAVDGQENPLAAVQAQKFYEVQKFLAMTNHILNDQLYLVSNETYKELPEDLQKVVKDAAENAAKYHTKLFVDGEKDLVTFFEKQGVKITHPDLVPFKESMKPYYAEFVKQTGQKGESALKQIEAINP
